# Ameliorative Effect of Oxytocin on *FBN1* and *PEPCK* Gene Expression, and Behavioral Patterns in Rats' Obesity-Induced Diabetes

**DOI:** 10.3389/fpubh.2022.777129

**Published:** 2022-04-07

**Authors:** Asmaa Elnagar, Khalifa El-Dawy, Hussein I. El-Belbasi, Ibrahim F. Rehan, Hamdy Embark, Zeinab Al-Amgad, Obeid Shanab, Elsayed Mickdam, Gaber E. Batiha, Salman Alamery, Samer S. Fouad, Simona Cavalu, Mohammed Youssef

**Affiliations:** ^1^Department of Biochemistry, Faculty of Veterinary Medicine, Zagazig University, Zagazig, Egypt; ^2^Department of Husbandry and Development of Animal Wealth, Faculty of Veterinary Medicine, Menofia University, Shebin Alkom, Egypt; ^3^Department of Physiology, Faculty of Veterinary Medicine, South Valley University, Qena, Egypt; ^4^General Authority for Veterinary Services, Ph.D in Veterinary Pathology and Clinical Pathology, Faculty of Veterinary Medicine, South Valley University, Qena, Egypt; ^5^Department of Biochemistry, Faculty of Veterinary Medicine, South Valley University, Qena, Egypt; ^6^Department of Nutrition and Clinical Nutrition, Faculty of Veterinary Medicine, South Valley University, Qena, Egypt; ^7^Department of Pharmacology and Therapeutics, Faculty of Veterinary Medicine, Damanhour University, Damanhour, Egypt; ^8^Department of Biochemistry, College of Science, King Saud University, Riyadh, Saudi Arabia; ^9^Qena University Hospital, Ph.D in Veterinary Clinical Pathology, South Valley University, Qena, Egypt; ^10^Department of Pharmacy, Faculty of Medicine and Pharmacy, University of Oradea, Oradea, Romania

**Keywords:** asprosin, *FBN1*, *PEPCK*, diabetes, motor activity

## Abstract

Amelioration of hyperinsulinemia and insulin resistance associated with obesity is a cardinal target for therapeutics. Therefore, we investigated the relation of *Fibrilln-1* (*FBN1) mRNA* expression and hepatic *phosphoenolpyruvate caboxykinase* (*PEPCK*) enzyme to the ameliorative impact of oxytocin on obesity-induced diabetes, suggesting glycogenolysis markers in diabetic models. Four groups of forty male Wistar rats were formed (*n* = 10): a control group fed basal diet and *intra*peritoneal injections of saline; an oxytocin-injected group; a diet-induced obese group fed a high-fat/high-sugar diet and injected with saline; a diet-induced obese group injected with oxytocin. Depending on blood glucose levels, obese groups were further sub-grouped into prediabetic, and diabetic rats, with 5 rats each, at the ninth and the 16th week of the feeding period, respectively. *FBN1* expression and *PEPCK* activity were determined using the *qPCR* technique and some biochemical parameters (glycemic, lipid profile, kidney, and liver functions) were determined using kits. Obese groups showed an elevation of brain *FBN1* expression, high serum lipid profile, high glucose level, and a deleterious impact on liver and kidney functions. Obese groups showed the stimulator effect of the *PEPCK* enzyme and time-dependent pathological changes in renal and hepatic tissues. The motor activities were negatively correlated with *FBN1* gene expression in prediabetic and diabetic rats. In addition to our previous review of the crucial role of asprosin, here we showed that oxytocin could ameliorate obesity-induced diabetes and decrease *FBN1* gene expression centrally to block appetite. Oxytocin caused decreases in *PEPCK* enzyme activity as well as glycogenolysis in the liver. Therefore, oxytocin has a potential effect on *FBN1* expression and *PEPCK* enzyme activity in the obesity-induced diabetic-rat model.

## Introduction

Type 2 diabetes mellitus (T2DM) influences the death toll among diabetic patients with pathological complications. Reports have shown that diabetes will show more prevalence as a major health concern in the next few decades (1). Thus, the introduction of new diagnostic biomarkers is crucial for effective therapy. Obesity has been linked to the development of T2DM, which is associated with insulin resistance and hyperinsulinemia (2). Recently, a newly discovered glucogenic hormone “asprosin” was enriched in white adipose tissue (WAT) and encoded by *Fibrillin1* gene (*FBN1*) (3). Asprosin consists of 140 amino acids it is a result of *profibrillin*-1's *C*-terminal cleavage via the activation of the *protease furin*. *FBN1 mRNA* expression showed a high level in WAT of humans and rats, and asprosin level is raised significantly during T2DM and the release of glucose from the liver, which suggests the crucial role of this adipokine in T2DM pathogenesis. It was, however, much lower in neonatal patients and Marfan Progeroid Syndrome because of a truncated mutation in the *FBN1* gene, meaning they had a limited appetite and were extremely thin (3).

There are two possible pathways of asprosin releasers to regulate body homeostasis; the first binds to the hepatocyte surface to initiate hepatic glucose release during glycogenolysis into the bloodstream, via G protein and cyclic adenosine monophosphate (cAMP)-dependent protein kinase A (PKA) axis pathway (4). Moreover, the second pathway is achieved through the crossing of circulatory asprosin through the blood-brain barrier and activation of orexigenic (AgRP^+^) neurons across a cAMP-dependant pathway, and inhibition of anorexigenic, Pro-opiomelanocortin^+^ (POMC^+^) neurons via a gamma-aminobutyric acid (GABA) dependant manner, leading to stimulation of appetite, gluconeogenesis, and adiposity (5). The liver thus manufactures glucose through two mechanisms: gluconeogenesis (synthesis of glucose) and glycogenolysis (enzymatic glycogen breakdown catalyzed by *glycogen phosphorylase*). During fasting, asprosin has a biological role *in vivo* because it enhances the liver to release glucose to maintain homeostasis, as well as central appetite stimulation. Asprosin levels are linked to glucose metabolism, obesity, lipid profiles, insulin resistance, renal function, and the functions of β-cells (6). Hence, T2DM is chronically followed by degeneration of β-cells, and regeneration is needed to cure diabetes.

Multifunctional 9-amino acid neuropeptide hormones and oxytocin synthesized in the hypothalamus share social behaviors including maternal bonding (7). In this regard, oxytocin has a therapeutic impact in diabetic subjects through induction of insulin secretion (8) and regeneration of β-cells (9). Meanwhile, prolonged oxytocin therapy was found to lower blood glucose levels in diabetic rats (10). In metabolic syndrome individuals with or without prediabetes, oxytocin is linked to glucose intolerance (11). Oxytocin stimulates glucose absorption in muscle cells, and the existence of oxytocin specific-receptors in the Langerhans of rats has validated its role in insulin and glucagon release (12). Both asprosin and glucagon hormones prevent blood glucose levels from lowering and therefore stimulate liver glycogenolysis. Additionally, the influence of oxytocin on insulin secretion is regulated centrally through vagal cholinergic neurons, which innervate ß-cells, and peripherally through inducing the activities of *phosphoinositide* and *protein kinase C* in ß-cells (13).

It has been noted that the treatment of asprosin exerts no impact on the levels of glucogenic hormones (14). It is also commonly known that the glucogenic effects of glucagon can also be achieved through activating the G protein-cAMP-PKA axis; inhibiting the receptor of glucagon, however, has no impact on the asprosin glucogenic influences on hepatocytes. This indicates asprosin plays a relatively independent role in promoting glucose release. As for appetite mediating effects, a study by the same team demonstrated the link between asprosin and ghrelin, a well-known orexigenic hormone released from the stomach. The findings revealed that a fractional overlapping subset of AgRP^+^ neurons could be activated by both asprosin and ghrelin. Furthermore, the deficiency of asprosin resulted in the reduced ability of ghrelin to activate AgRP^+^ neurons.

The Ghrelin receptor, on the other hand, is dispensable for the ability of asprosin to activate AgRP^+^ neurons. Oxytocin reduces body weight growth while increasing motor activity (15) by enhancement of lipolysis, and fatty acids β-oxidation. This is most likely due to increased acute insulin production, which aids glucose mobilization across cell membranes and subsequently decreases blood glucose levels. To prove oxytocin's value as a therapeutic target, it is necessary to clarify whether oxytocin can boost the first phase of insulin secretion in the initial stage of diabetes (9). Also, insulin enhances glycogen synthesis and blocks gluconeogenesis and glycogenolysis through *post*-translational modification of *phosphoenolpyruvate carboxykinase* (*PEPCK*) enzyme and its gene expression (16). Therefore, the conversion of *oxaloacetate* to *phosphoenolpyruvate* is catalyzed by such potential effect of the *PEPCK* enzyme in order to regulate the glucose level through glycogenolysis in diabetic patients (17). On a behavioral basis, oxytocin modulates neuroendocrine reflexes for the regulation of learning and memory.

For the present study, we hypothesized that the potential impact of oxytocin on glucose level and insulin might be mediated by *FBN1* expression and *PEPCK* enzymatic activities in obese diabetic rats. Therefore, this evaluated the effect of oxytocin administration on some biochemical parameters, molecular, pathological, and motor activity profiles of diabetic rats, in relation to *FBN1* gene expression.

## Materials and Methods

### Animal Management

This study used forty-mature male Wistar rats weighing an average of 260 ± 19.2 g and aged 7–8 weeks. They were obtained from the Faculty of Veterinary Medicine, Sohag University, Sohag, Egypt. Animals were acclimatized for 14 days in a laboratory before the beginning of the experiment. They were kept at room temperature with a normal 12 Light/12 Dark cycle and a free source of food and drink. All animals were carefully managed and reared during optimal weather conditions in addition to ensuring a low-stress environment to maximize their welfare.

### Study Design

After 2 weeks from adaptation, the experimental design involved four groups (10 rats each), as follows:

- Group A; the control group was given a standard diet with an *intra*peritoneal injection of saline (Control).
- Group B; were given a standard diet and injected with oxytocin (Control + OX).
- Group C; were given a high-fat high sugar diet (HFHSD), and injected with saline.
  ° Group C1: (Prediabetic + saline).
  ° Group C2: (Diabetic + saline).
- Group D; were given HFHSD and injected with oxytocin “OX”.
  ° Group D1: (Prediabetic + OX).
  ° Group D2: (Diabetic + OX).

Depending on glucose level, each treated group of obese rats was further sub-divided into 2 sub-groups with 5 rats as prediabetic and the rest as diabetics. Two types of ration were prepared freshly and daily throughout the experiment; (1) control normal diet, and (2) high-fat high sugar diet (HFHSD). Rats in the control group were given a typical standard diet with no additives, including the following: 22.8% carbohydrates, 25.8 proteins, and 11.4% fat, 12.6 KJ/g in total (18).

For seven days at the 9th and 16th week of the experiment, 1 mL of saline solution was injected intraperitoneally every day. The composition of HFHSD was as follows: (22.6% carbohydrates including 10% sucrose in drinking water, 16.4% protein, 58% fat “soybean oil or pork fat”), 23.4 KJ/g in total for the period of the experiment. We used oxytocin (syntocinon®, Novartis LTD., 10 IU/mL, 16.7 μg/mL) as a therapeutic hormone of diabetes; twice *intra*-peritoneal injection with oxytocin 2 mg/kg body weight (anti-obesity and anti-diabetes) was given once a day for seven days at night on the 9th and 16th week of the experiment. After the rats were fed the HFHS diet, the 9th week was the expected time of them being prediabetic, while the 16th week was the time of diabetes (19).

The last dose was divided into two doses: one was at night and the second was on the morning of the end of the 9th and 16th week, for the biochemical analysis. Rats were fasted overnight at the end of the 9th week and the 16th week immediately after the last dose of oxytocin injection. They were then sacrificed through a cervical decapitation while being anesthetized under the effect of ether at the end of the experiment. It is reported that HFHSD induced diabetes in rats after 12 weeks duration (20). However, we continued the experiment until 16 weeks to ensure the influences of diabetes-associated obesity on the lipid profiles and liver and kidney functions.

### Animals' Weights

Before the supplementation of different diets' treatment and oxytocin, the initial body weights were recorded for each rat. Afterward, the body weights were taken weekly and the final body weights were taken just before the sacrifice of animals.

### Assessment of Diabetic Condition

Fasting plasma glucose (FPG) level was assessed after the 9th and 16th week of different diets using the glucose oxidase method by Spectrum diagnostic kits (21). Rats with a glucose level between 105 and 126 mg/dl were considered prediabetic; while diabetic rats were those who had a blood glucose level of more than 126 mg/dl (22).

### Plasma and Serum Collection

Blood was collected from all rats, and plasma is the liquid that remains when clotting is prevented with the addition of an anticoagulant (0.109 M sodium citrate). Nine parts of freshly collected whole blood should be immediately added to one part of the anticoagulant. Then, the blood was centrifuged at 2,500 × g for 15 min. Separate the plasma using a plastic pipette and place it in a plastic test tube. Perform the Prothrombin Time assay within 4 h which was stored at −20°C until used for the estimation of glucose level (mg/dl). Reconstitute the control plasmas (normal control plasma, abnormal control plasma) according to the package insert included with the control.

### Biochemical Analyses

#### Biochemical Colorimetric Assays

The biochemical analyses were performed immediately at the time of sampling collection. Sera were collected and kept after the blood has clotted for creatinine (mg/dl), albumin (g/dl), alanine aminotransferase (ALT) (U/L), aspartate aminotransferase (AST) (U/L), cholesterol (mg/dl), high-density lipoprotein (HDL-c) (mg/dl), low-density lipoprotein (LDL-c) (mg/dl), very low-density lipoprotein (VLDL-c) (mg/dl), and total triacylglycerol (TAG) (mg/dl). Serum LDL-c calculation was determined using Friedewald's formula (23), as the following equation: Serum LDL-c (mg/dl) = TC (mg/dl)–[HDL-c (mg/dl) + TAG (mg/dl)/5], whereas VLDL-c (mg/dl) = TAG/5. All these biochemical parameters were detected using related commercial kits (Spectrum, Egypt), and assays were performed as per the manufacturer's guidelines; insulin resistance can be measured using the homeostatic model assessment of insulin resistance (HOMA-IR). The following equation was used to calculate HOMA-IR: [HOMA-IR = fasting plasma glucose (mg/dl) X fasting insulin (mIU/L)/405) (24).

#### ELISA

As per the manufacturer's guidelines, the serum insulin level was determined using the RAT_(ins)_ ELISA kit^TM^ (SunRed China, Shanghai), according to the previous method (24). In a plate with monoclonal insulin antibody pre-coated, biotin-labeled and streptavidin-HRP-combined antibodies were added to serum samples, and the reaction was allowed to run for 60 min at 37°C. The plate was rinsed five times before being exposed to chromogen solutions for 10 min at 37°C. After 10 min, the absorbance was measured after the stop solution was applied. The standard curve's linear regression equation was used to determine an unknown insulin concentration.

### Molecular Analyses

#### qPCR and RNA Extraction

GENEzol^TM^ reagent (Taiwan, New Taipei) was used to extract *RNA* from tissue samples (25). Biopsies from the brain and liver (50–100 mg each) were homogenized in a few minutes using tissue glass Teflon in 1 ml of GENEzol^TM^ reagent, and then the *RNA* aqueous phase was then separated with care using chloroform. Isopropanol was used to precipitate *RNA*, which was then rinsed with 75 percent ethanol. The *RNA* pellet was cleaned and air-dried before being redissolved in DPEC-treated water. Using the Topreal^TM^ One-step RT *qPCR* kit, 2 μg *RNA* was reverse-transcribed to *cDNA* and duplicated (Enzynomics, Daejeon, Republic of Korea). The expression of *mRNA* was assessed using the ΔΔCt method and represented relatively to *GAPDH* as an endogenous control. The primers (26) used for qPCR reaction were as follows;

° *GAPDH* sense: 5'-CAGCAATGCATCCTGCAC-3' and antisense: 5'-GAGTTGCTGTTGAAGTCACAGG-3'. The gene bank accession number is (XM_017592435.1), the annealing temperature (Ct) is 26.2–27.7°C, and the melting temperature is 72–73°C.
° *Neural FBN1* sense: 5'-GAGTGTGAACTGAGCGCGA-3' and antisense 5'-AGGCACACTCGTACTTCCCA-3'. The gene bank accession number is (NM_000138), the annealing temperature (Ct) is 32.3–34.3°C, and the melting temperature is 74.5–76°C.
° Hepatic *PEPCK* sense: 5'-GTCACCATCACTTCCTGGAAGA-3' and antisense: 5'-GGTGCAGAATCGCGAGTTG-3'. The gene bank accession number is (AH_007109), the annealing temperature (Ct) is 30–35.7°C, and the melting temperature is 80–80.5°C.

### Motor Behavior

The motor activities of the rats were assessed using Panlab IR Actimeter Harvard Apparatus (27). The activity was recorded using transparent boxes supplied by upper and lower two-dimensional square infrared frames. The upper frame is used for recording rearing activity while the lower frame was recording the general activity, locomotor, and stereotypic behavior of rats. The positions of frames were estimated depending on rats' size with the upper edge of the lower, and the upper frame was 4 and 14.5 cm high from the box floor, respectively.

### Pathological Analyses

#### Organs Collection

Pancreas, liver, and kidney were dissected from all groups. The dissected organs were followed by fixation in 10% buffered formaldehyde.

#### Tissue Preparation and Staining

##### Preparation of Samples

As soon as the animals were sacrificed, the pancreas, liver, and kidney were dissected from all groups. The dissected organs were immediately checked for any gross lesions or macroscopic changes. The dissected organs were fixed in 10% buffered formaldehyde.

##### Procedures of Staining and Microscopic Monitoring

After organ fixation in 10% buffered formaldehyde, standard histology techniques were used to process it by running it through various grades of ethyl alcohol (70, 80, 90, and 100 percent). Then, clearance occurred in xylene and was embedded in paraffin wax. Hematoxylin and eosin (HE) were used to stain five-micrometer-thick serial slices for light microscopy (28).

### Statistical Analysis

A one-way ANOVA test was used to compare the means of the different groups, followed by Tukey's posthoc test. For importing, modifying, and applying statistical analysis of data, SPSS for statistical computing (29) was utilized. The results were calculated using the formula [mean + standard error mean (SEM)]. When the *P* < 0.05, the means were judged statistically different. *Pearson's Correlation Coefficients* of biochemical markers and the motor activities of samples with genetic expression were displayed using *GraphPad Prism*.

## Results

Diabetic-associated obesity is a metabolic disorder caused by changes in insulin secretion and characterized by persistent hyperglycemia and disturbances in carbohydrate, protein, and lipid metabolism. This study is the first to clarify the influence of the asprosin gene (*FBN1*) and *PEPCK* enzyme activity on diabetic rats and after oxytocin therapy.

### Rats' Body Weights and Glucose Level Before and After Giving Oxytocin Injection

**Figure 1:**
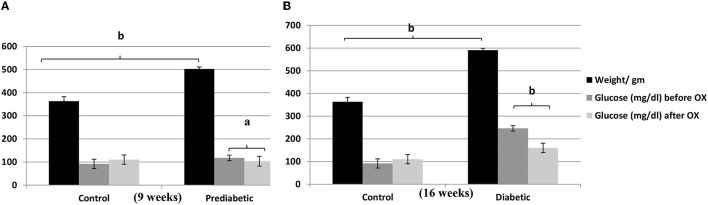
Relationship of the weights (gm ± SEM) of Wistar rats with the glucose level (mg/dl ± SEM) before and after oxytocin injection; **(A)** at 9th week, and **(B)** at 16th week after diet treatments. ^a^*P* ≤ 0.05 and ^b^*P* ≤ 0.001, OX, oxytocin.

There is a strong relationship between the weights (gm) of rats and the glucose level (mg/ dl) before and/or after oxytocin injection at the 9th week of prediabetics (Figure 1A) and week 16 of diabetics (Figure 1B) after HFHSD comparable to the control Wistars. The bodyweight of prediabetic and diabetic rats increased with significance differences [*P* = 0.006, *P* = 0.001, *F*_(3, 36)_ = 54.23] compared to controls. The present research confirmed that in the control group injected with oxytocin, the glucose release was reduced significantly [*P* = 0.044 in prediabetics, and *P* = 0.007 in diabetics, *F*_(3, 36)_ = 29.13] compared to those in the untreated prediabetic group.

### Bodyweight Curve in Control and Diabetic-Obese Rats

**Figure 2:**
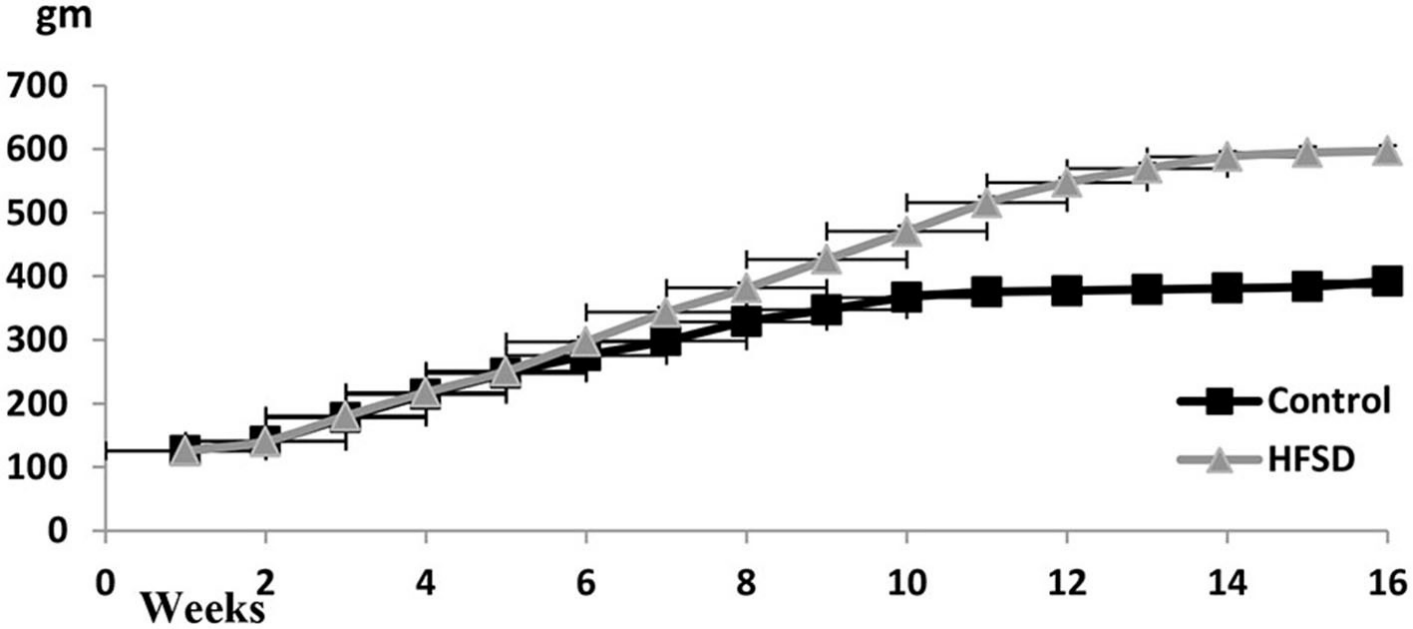
Body weights (gm ± SEM) of Wistar rats from the beginning of the experiment until the 16th week. The prediabetic rat was determined on the 9th week and the diabetic determined on the 16th week. HFHSD: high-fat high sugar diet.

The bodyweight curve along with the experimental duration until week 16 was ascending, particularly in the case of HFHSD treatment (Figure 2).

### Glycemic Markers of Control and Diabetic-Obese Rats

We analyzed some of the glycemic markers, such as glucose (mg/dl), insulin (mIU/L), and HOMA-IR. For instance, in Table 1, oxytocin diminishes the glucose level in prediabetic and diabetic rats with significances [*P* = 0.037, *P* = 0.004, respectively, *F*_(3, 36)_ = 26.48] compared to untreated ones. The same effect of oxytocin had been achieved for decreasing insulin in prediabetic and diabetic rats but with significances [*P* = 0.008, *P* = 0.007, respectively, *F*_(3, 36)_ = 29.41] compared to the untreated groups. Meanwhile, the obese-prediabetic and obese-diabetic groups had substantially higher HOMA-IR [*P* = 0.007, *P* = 0.003, *F*_(3, 36)_ = 46.33] than control rats.

**Table 1:** Effect of oxytocin administration on serum glucose, insulin conc., and HOMA-IR in different groups of Wistar rats.

**Parameters**	**Control** **(normal)**	**Control + OX** **(normal)**	**Pre-diabetic** **(HFHSD)**	**Pre-diabetic + OX** **(HFHSD)**	**Diabetic** **(HFHSD)**	**Diabetic + OX** **(HFHSD)**	**Significance** **(*P*-values)**
Glucose (mg/dl)	92.00 ± 8.20	91.50 ± 10.50	117.83 ± 12.11	103.81 ± 11.43^a^	246.22 ± 19.11	160.45 ± 11.1^c^	•^a^*P* = 0.037 •^c^*P* = 0.004
Insulin (mIU/L)	25.45 ± 7.35	20.01 ± 7.22	26.02 ± 3.29	23.33 ± 3.44^b^	27.12 ± 2.10	24.54 ± 3.6^c^	•^b^*P* = 0.008 •^c^*P* = 0.007
HOMA-IR	5.78 ± 7.35	7.09 ± 7.35	8.73 ± 1.35	6.49 ± 1.11^b^	19.52 ± 2.35	9.72 ± 2.35^c^	•^b^*P* = 0.007 •^c^*P* = 0.003

*The data are represented as (mean ± SEM). HOMA-IR, homeostatic model assessment of insulin resistance; HFHSD, high-fat-high-sugar diet; OX, oxytocin; and SEM, standard error mean*.

a *P < 0.05 in comparison with prediabetic group*.

b *P < 0.01 in comparison with prediabetic group*.

c *P < 0.01 in comparison with diabetic group*.

### Hepatic Enzymes and Lipid Profiles of Control and Diabetic-Obese Rats

We analyzed the hepatic enzyme (Table 2). Serum ALT (U/L) was decreased in prediabetic and diabetic groups significantly after being treated with oxytocin [*P* = 0.05, *F*_(3, 36)_ = 52.23, *P* = 0.022, respectively, *F*_(3, 36)_ = 54.16] compared to untreated groups. Similarly, serum AST (U/L) was also decreased after oxytocin treatment in prediabetic and diabetic groups significantly after being treated with oxytocin [*P* = 0.008, *P* = 0.007, respectively, *F*_(3, 36)_ = 37.13] compared to untreated groups.

**Table 2:** Comparison (mean ± SEM) of the hepatic enzymes and the lipid profiles in different groups of Wistar rats.

**Parameters**	**Control** **(normal)**	**Control + OX** **(normal)**	**Pre-diabetic** **(HFHSD)**	**Pre-diabetic + OX** **(HFHSD)**	**Diabetic** **(HFHSD)**	**Diabetic + OX** **(HFHSD)**	**Significance** **(*P*-values)**
ALT (U/L)	23.54 ± 4.66	29.31 ± 1.82	31.15 ± 3.94	28.74 ± 1.14^b^	41.64 ± 2.53	39.47 ± 2.87^e^	•^b^*P* = 0.043 •^e^*P* = 0.032
AST (U/L)	62.86 ± 18.90	61.17 ± 23.75	139.35 ± 21.19	129.54 ± 17.98^c^	160.82 ± 12.61	152.31 ± 15.46^f^	•^c^*P* = 0.008 •^f^*P* = 0.007
HDL-c (mg/dl)	43.11 ± 5.32	42.44 ± 5.21	45.22 ± 6.23	43.78 ± 3.66^b^	47.33 ± 3.88	45.38 ± 3.55^e^	•^b^*P* = 0.044 •^e^*P* = 0.038
LDL-c (mg/dl)	29.23 ± 4.43	26.32 ± 2.66	51.27 ± 4.22	48.66 ± 3.22^c^	67.31 ± 4.11	51.29 ± 4.88^f^	•^c^*P* = 0.007 •^f^*P* = 0.005
TC (mg/dl)	72.34 ± 8.74	68.76 ± 9.60^a^	96.49 ± 6.00	92.34 ± 5.15^c^	114.64 ± 5.44	96.67 ± 4.08^f^	•^a^*P* = 0.048 •^c^*P* = 0.007 •^f^*P* = 0.006
TAG (mg/dl)	64.11 ± 3.86	42.12 ± 3.52^a^	85.87 ± 3.34	58.51 ± 3.02^d^	177.82 ± 25.23	136.23 ± 8.14^g^	•^a^*P* = 0.0044 •^d^*P* = 0.000 •^g^*P* = 0.001
VLDL-c (mg/dl)	12.82 ± 1.23	8.42 ± 1.11	17.17 ± 2.43	11.70 ± 1.55^c^	35.56 ± 3.77	27.24 ± 2.69^f^	•^c^*P* = 0.008 •^f^*P* = 0.006

*The data is represented as (mean ± SEM). ALT, alanine transaminase; AST, aspartate transaminase; HDL-c, high-density lipoprotein cholesterol; LDL-c, low-density lipoprotein cholesterol; TAG, triacylglycerol; TC, total cholesterol; VLDL-c, very low-density lipoprotein cholesterol; HFHSD, high-fat-high-sugar diet; OX, oxytocin; and SEM, standard error mean*.

a *P < 0.05 in comparison with control group*.

b *P < 0.05 in comparison with pre-diabetic group*.

c *P < 0.01 in comparison with pre-diabetic group*.

d *P < 0.001 in comparison with pre-diabetic group*.

e *P < 0.05 in comparison with diabetic group*.

f *P < 0.01 in comparison with diabetic group*.

g *P < 0.001 in comparison with diabetic group*.

We analyzed the lipid profiles in sera [HDL-c (mg/dl), LDL-c, VLDL-c (mg/dl), TAG (mg/dl), TC (mg/dl)]. The cholesterol was analyzed (Table 2), HDL-c was significantly improved after oxytocin injection in prediabetic and diabetic groups [*P* = 0.044, *P* = 0.038, respectively, *F*_(3, 36)_ =30.77] compared to those that were untreated. The LDL-c was decreased in treated prediabetic and diabetic groups with significance [*P* = 0.007, *P* = 0.005, respectively, *F*_(3, 36)_ = 47.15] compared to untreated ones. In parallel, VLDL-c was decreased in treated prediabetic and diabetic with significances [*P* = 0.008, *P* = 0.006, respectively, *F*_(3, 36)_ = 49.53] compared to untreated ones. Thus, in the presence of oxytocin medication, the TC in all treated groups reduced [*P* = 0.048, *P* = 0.007, and *P* = 0.006, in control, prediabetic, and diabetic, respectively; *F*_(3, 36)_ = 28.14] compared to those of treated rats. Moreover, oxytocin plays a big role in lowering the TAG levels in control, prediabetic, and diabetic rats [*P* = 0.004, *P* = 0.000, and *P* = 0.001, respectively, *F*_(3, 36)_ = 29.13] compared to those untreated groups.

### Serum Creatinine and Albumin of Control and Diabetic-Obese Rats

We analyzed creatinine (mg/dl) and albumin (g/dl) in rat sera. As shown in Table 3, creatinine was decreased in normal, prediabetic, and diabetic rats after they were given oxytocin with significance values [*P* = 0.046, *P* = 0.027, and *P* = 0.04, respectively, *F*_(3, 36)_ = 30.14] compared to those that were untreated. However, the albumin level was increased in prediabetic and diabetic rats after oxytocin administration [*P* = 0.047, *P* = 0.031, respectively, *F*_(3, 36)_ = 38.13] compared to untreated rats.

**Table 3:** Alterations (mean ± SEM) of creatinine and albumin in different groups of Wistar rats.

**Parameters**	**Control** **(normal)**	**Control + OX** **(normal)**	**Pre-diabetic** **(HFHSD)**	**Pre-diabetic + OX** **(HFHSD)**	**Diabetic** **(HFHSD)**	**Diabetic + OX** **(HFHSD)**	**Significance** **(*P*-values)**
Creatinine (mg/dl)	0.78 ± 0.33	0.70 ± 0.78^a^	1.15 ± 0.59	0.90 ± 0.20^b^	1.08 ± 0.42	0.87 ± 0.18^c^	•^a^*P* = 0.046 •^b^*P* = 0.027 •^c^*P* = 0.04
Albumin (g/dl)	3.81 ± 0.32	4.00 ± 0.22	3.61 ± 0.25	3.82 ± 0.35^b^	3.51 ± 0.27	3.71 ± 0.21^c^	•^b^*P* = 0.047 •^c^*P* = 0.039

*The data are represented as (mean ± SEM). HFHSD, high-fat-high-sugar diet; OX, oxytocin; and SEM, standard error mean*.

a *P < 0.05 in comparison with control group*.

b *P < 0.05 in comparison with prediabetic group*.

c *P < 0.05 in comparison with diabetic group*.

### Motor Activity of Control and Diabetic-Obese Rats

The motor activities/sec of rats in all groups were observed (Table 4), in which the oxytocin treatment improved the activities of rats. For instance, in control-treated oxytocin the locomotion, general activity, distance, stereotypic behavior, rearing, and fast movement were increased significantly [*P* = 0.0074, *F*_(3, 36)_ = 30.16; *P* = 0.000, *F*_(3, 36)_ = 22.32; *P* = 0.0054, *F*_(3, 36)_ = 36.14; *P* = 0.0064, *F*_(3, 36)_ = 32.52; *P* = 0.043, *F*_(3, 36)_ = 40.13; and *P* = 0.0043, *F*_(3, 36)_ = 41.66, respectively], compared to the untreated controls. Moreover, in diabetic treated oxytocin rats recorded [*P* = 0.006, *F*_(3, 36)_ = 29.54; *P* = 0.001, *F*_(3, 36)_ = 20.22; *P* = 0.043, *F*_(3, 36)_ = 34,66; *P* = 0.005, *F*_(3, 36)_ = 30.01; *P* = 0.006, *F*_(3.36)_ = 38.32; and *P* = 0.043, *F*_(3, 36)_ = 39.13, respectively], compared to the diabetics.

**Table 4:** Motor activity/sec (mean ± SEM) in different groups of Wistar rats.

**Motor activities/sec**	**Control (normal)**	**Control + OX (normal)**	**Diabetic (HFHSD)**	**Diabetic + OX (HFHSD)**	**Significance (*P*-value)**
Locomotion	613.56 ± 75.41	873.00 ± 76.88^b^	514.78 ± 44.22	744 ± 53.42^f^	•^b^*P* = 0.0074 •^f^*P* = 0.0061
General activity	668.22 ± 80.96	941.33 ± 80.32^c^	553.11 ± 52.23	796.33 ± 57.53^g^	•^c^*P* = 0.000 •^g^*P* = 0.001
Distance	992.38 ± 148.05	1,569.04 ± 206.57^b^	782.19 ± 90.74	1,166.38 ± 99.5^f^	•^b^*P* = 0.0054 •^f^*P* = 0.043
Stereotypic behavior	54.67 ± 5.96	68.33 ± 3.46^b^	38.33 ± 4.15	52.33 ± 4.17^f^	•^b^*P* = 0.0064 •^f^*P* = 0.0053
Resting time	131.93 ± 17.02	86.84 ± 9.85^b^	152.51 ± 11.53	103.42 ± 11.24^f^	•^b^*P* = 0.0075 •^f^*P* = 0.0067
Slow movement	87.02 ± 4.57	89.84 ± 5.17	87.6 ± 6.61	92.18 ± 1.39^f^	•^f^*P* = 0.0081
Number of rearing	17.22 ± 2.03	30.00 ± 2.65^a^, ^d^	8.67 ± 1.42	22.22 ± 2.47^f^	•^a^*P* = 0.043, •^d^*P* = 0.036 •^f^*P* = 0.0062
Fast movement	81.04 ± 13.73	123.31 ± 11.84^b^	59.89 ± 8.88	104.40 ± 10.26^e^	•^b^*P* = 0.004 •^e^*P* = 0.043

*The data is represented as (mean ± SEM). HFHSD, high-fat-high-sugar diet; OX, oxytocin; SEM, standard error mean*.

a *P < 0.05 in comparison with control group,*

b *P < 0.01 in comparison with control group,*

c *P < 0.001 in comparison with control group,*

d *P < 0.001 in comparison with diabetic group,*

e *P < 0.05 in comparison with diabetic group,*

f *P < 0.01 in comparison with diabetic group,*

g *P < 0.001 in comparison with diabetic group*.

### Relative *FBN1 mRNA* Expression in Control and Diabetic Rats

**Figure 3:**
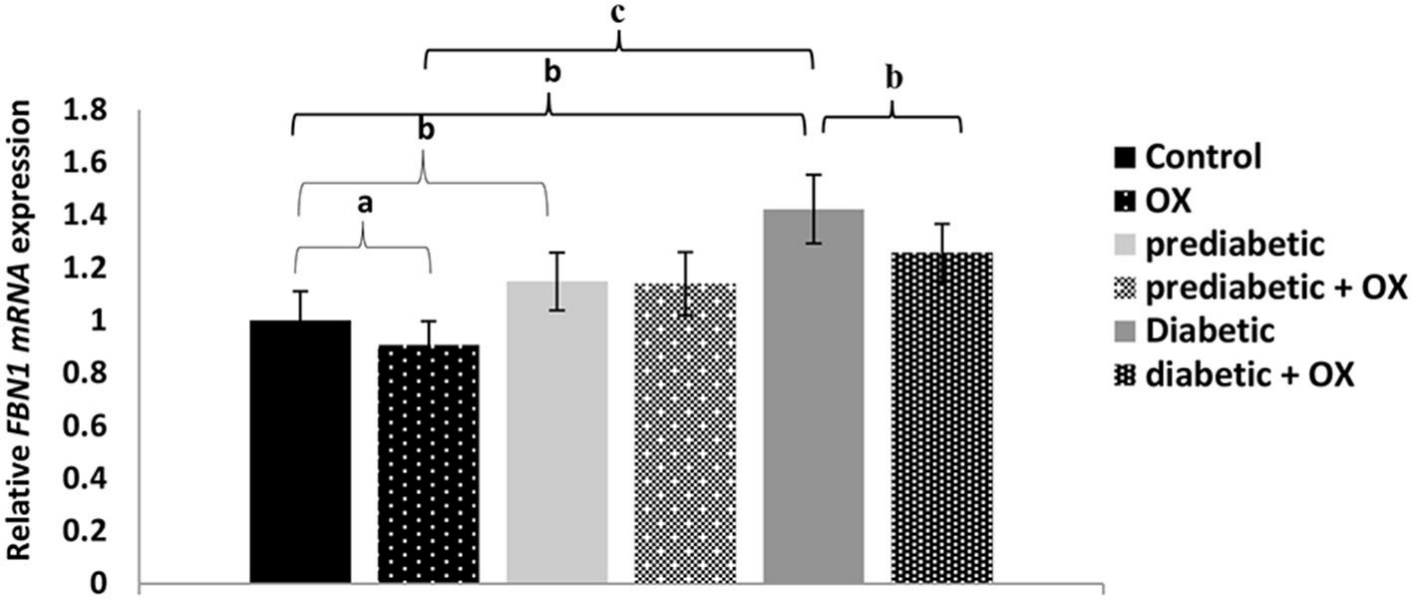
Relative *FBN1 mRNA* expression (mean ± SEM) in different groups of Wistar rats. *FBN1, fibrillin-1* gene; OX, oxytocin. ^a^*P* > 0.05, ^b^*P* > 0.01, and ^c^*P* > 0.001.

The relative *FBN1 mRNA* expression in different treated groups of Wistar rats is shown in Figure 3. As a result, we found a relationship between glucose level, *FBN1* expression, and oxytocin administration. For instance, the *FBN1* gene expression was increased in diabetes progress given statistical differences between control and prediabetic [*P* = 0.006, *F*_(3, 36)_ = 30.11]. It was also decreased significantly after oxytocin treatment in control and diabetic animals (*P* = 0.042, *P* = 0.008, respectively), compared to those who have not been treated. Given the highest expression of *FBN1* in the diabetic group with a high significance value (*P* = 0.000).

### Effect of Oxytocin on *PEPCK* Enzyme Gene in Control and Diabetic Rats

**Figure 4:**
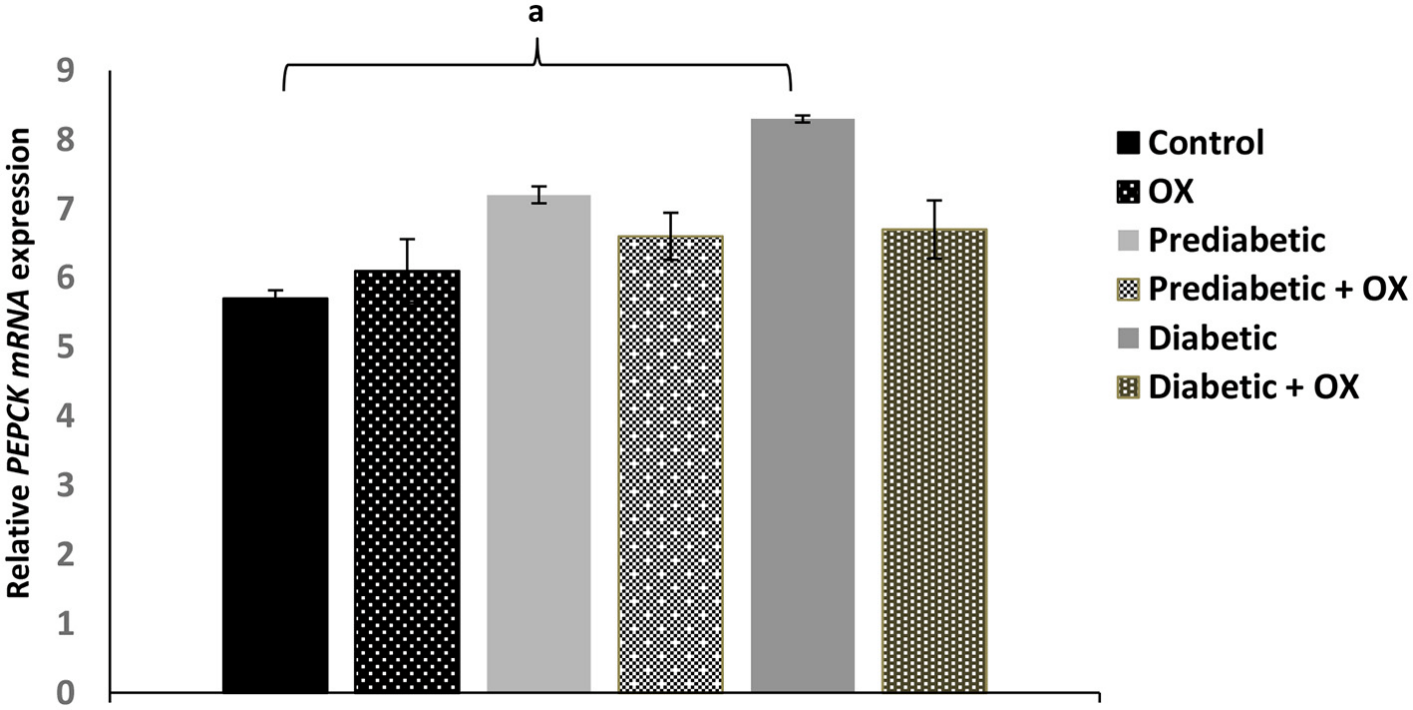
Effect of oxytocin medication on hepatic *PEPCK* enzyme activity (mean ± SEM) in different groups of Wistar rats. *PEPCK, phosphoenolpyruvate caboxykinase*, OX, oxytocin. ^a^*P* ≤ 0.05.

As shown in Figure 4, the relative *PEPCK mRNA* expression level was considerably enhanced [*P* = 0.042, *F*_(3, 36)_ = 28.19] in obese diabetic rats comparable to normal animals was significantly reduced, but also reduced in diabetic-treated animals with oxytocin with no statistical significances.

### Pathological Findings of the Pancreas in Control and Diabetic Rats

**Figure 5:**
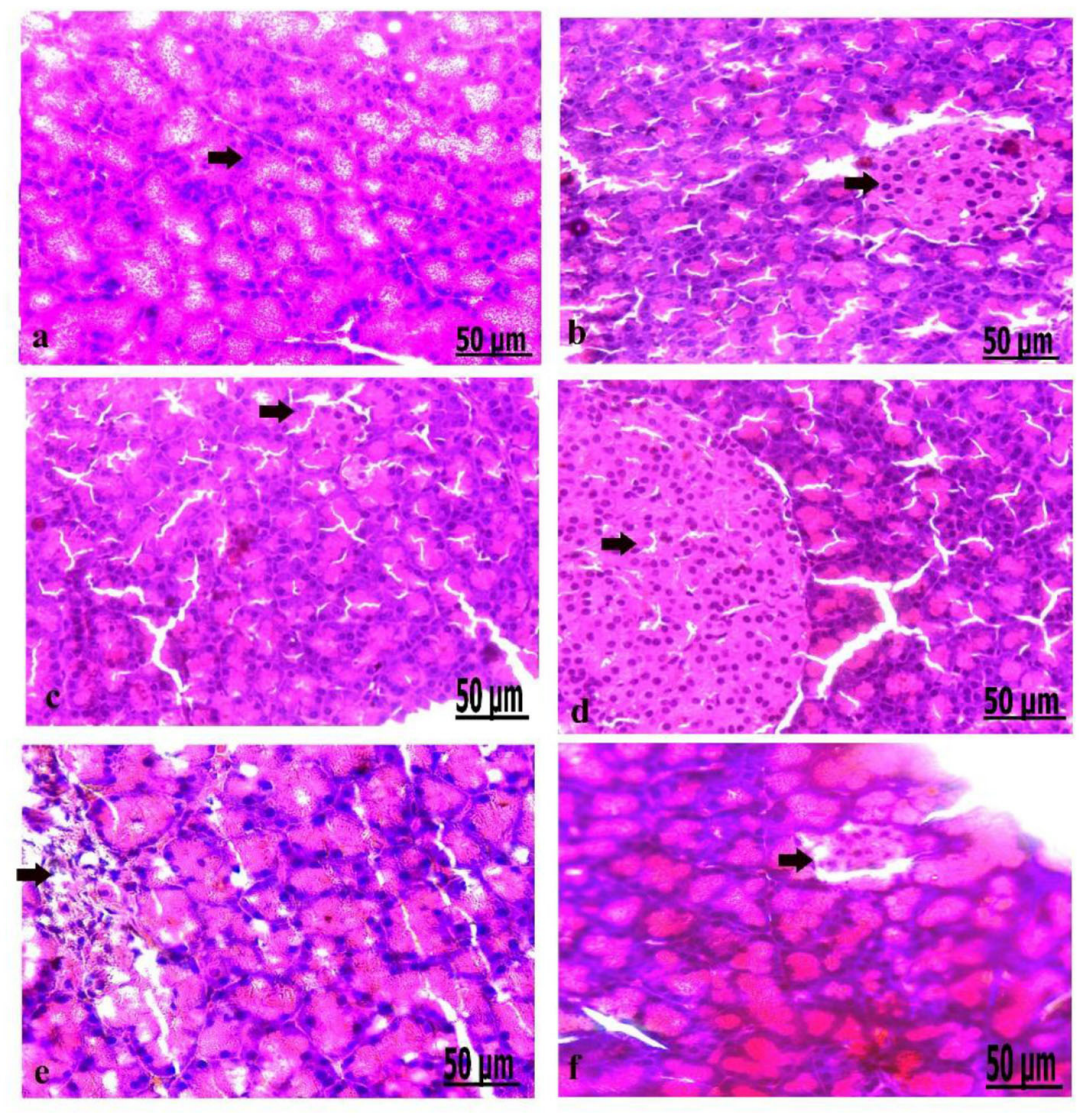
**(a–f)** Photomicrograph of the pancreas of the control group showed the structure of the pancreatic tissues comprising normal β-cells **(a)**. Photomicrograph of the pancreas of oxytocin group showed normally arranged pancreatic acini **(b)**. Photomicrograph of the pancreas of group 3 A (prediabetic non-treated) showed slight vacuolation of pancreatic acini and β*-*cells **(c)**. Photomicrograph of the pancreas of group 3 B (diabetic non-treated) showed mild vacuolation of β*-*cells of the Langerhans islets **(d)**. Photomicrograph demonstrated that the pancreas of group 4 A (prediabetic treated) showed a moderate degree of vacuolation and the islets of Langerhans **(e)**. Photomicrograph of the pancreas of group 4 B (diabetic treated) showed slight vacuolation of the islets of Langerhans **(f)** (H&E., bar = 50 μm).

On a pathological basis, we analyzed the pancreas (Figure 5). Macroscopically, gross lesions of pancreases of the control and oxytocin-treated control groups were normal. The pancreas of groups 3 A (prediabetic non-treated) and 3 B (diabetic non-treated) showed as normal. Moreover, in groups 4 A and B (prediabetic treated, and diabetic treated, respectively), we detected the normal appearance of the pancreas. In the liver, the control, and oxytocin treated groups showed normal appearance with normal shapes, sizes, and consistency. Group 3 A (prediabetic non-treated) showed reddish spots of congestion at liver capsules, besides the liver of group 3 B (diabetic non-treated) appeared pale in color. Group 4 A and B (prediabetic treated and diabetic treated, respectively), displayed slight congestion of the liver. In parallel, kidneys results in the control and oxytocin groups displayed normal cortex and medulla. Group 3 A (prediabetic non-treated) and group 3 B (diabetic non-treated) appeared small and had shrinkage, and suffered from darkish coloration due to a noticeable degree of congestion of the kidney. Groups 4 A and B (prediabetic treated and diabetic treated, respectively), displayed moderate congested cortex and medulla.

Microscopically, the pancreatic parenchyma in the control group had a normal histological structure, with pancreatic acini and normal Langerhans islet-cells (Figure 5a). The pancreas of oxytocin treated control group detected normally arranged pancreatic acini (Figure 5b). Group 3 A (prediabetic non-treated) recorded slight vacuolation of pancreatic acini and β-cell (Figure 5c). Group 3 B (diabetic non-treated) revealed mild vacuolation of β-cells in the Langerhans islets (Figure 5d). Moreover, the pancreas of group 4 A (prediabetic treated) showed a moderate degree of vacuolation of islets of Langerhans and pancreatic acini (Figure 5e). Group 4 B (diabetic treated) displayed slight vacuolation of the islets of Langerhans (Figure 5f).

### Pathological Findings of the Liver in Control and Diabetic Rats

**Figure 6:**
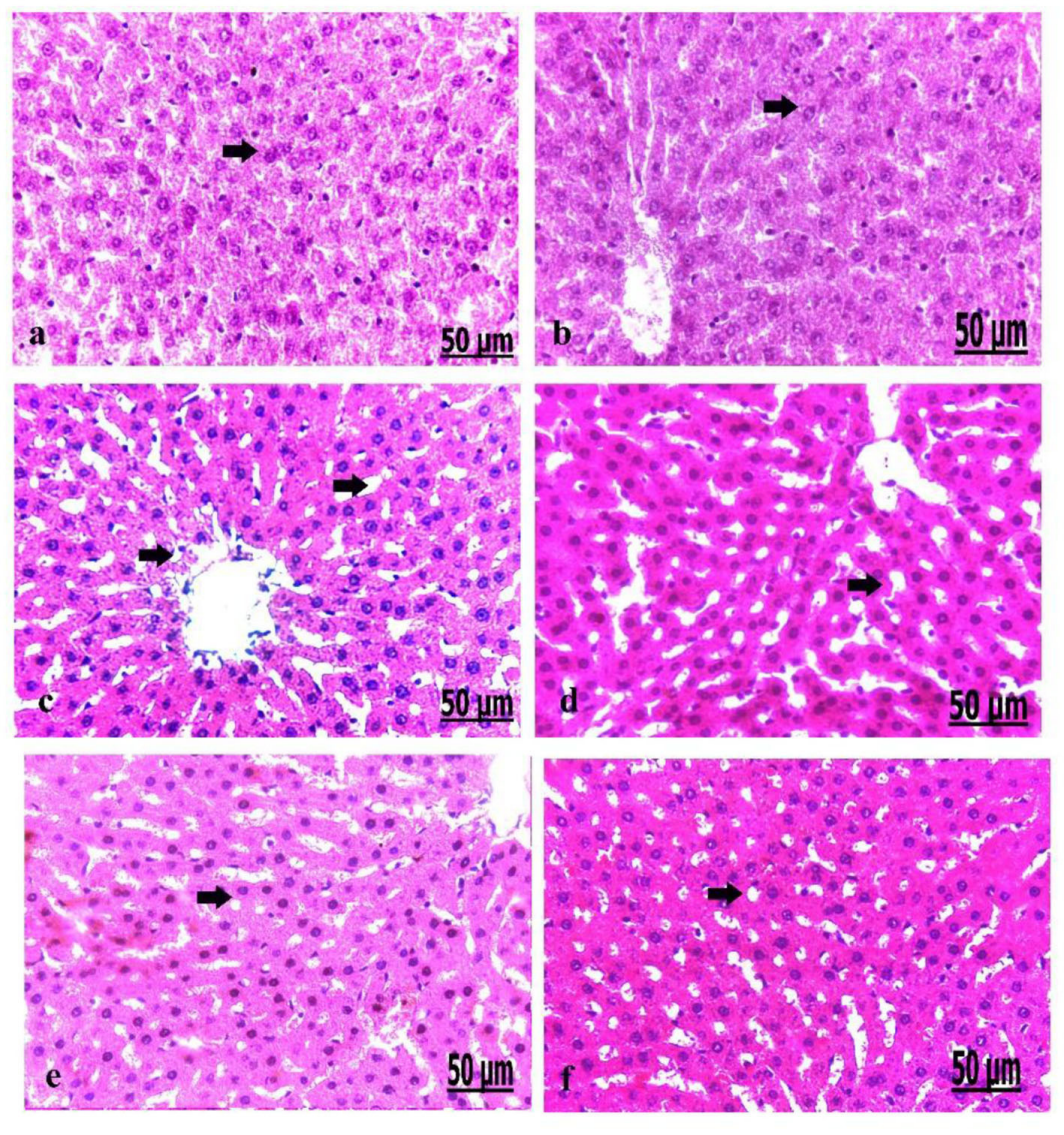
**(a–f)** Photomicrograph of liver of the control group showed normally arranged hepatocytes with normal vasculature **(a)**. Photomicrograph of the liver of oxytocin group showed normally arranged hepatocytes **(b)**. Photomicrograph of the liver of group 3 A (prediabetic non-treated) showed necrosis and fatty degeneration of hepatocytes with dilatation of the blood vessels **(c)**. Photomicrograph of the liver of group 3 B (diabetic non-treated) showed intensive necrosis and fatty degeneration of hepatocytes **(d)**. Photomicrograph of the liver of group 4 A (prediabetic treated) showed a mild degree of fatty degeneration and dilatation of the blood sinusoids **(e)**. Photomicrograph of liver of group 4 B (diabetic treated) showed fatty degeneration of hepatocytes with slight dilatation of the blood sinusoids **(f)** (H&E., bar = 50 μm).

We analyzed the liver on a pathological basis (Figure 6). The microscopic findings of the liver of the control group showed normally arranged hepatocytes with normal vasculature comprising normal blood sinusoids and bile duct (Figure 6a). The liver of the oxytocin treated control group showed normally arranged hepatocytes with a mild degree of dilatation of blood sinusoids (Figure 6b). The liver of group 3 A (prediabetic non-treated) showed necrosis and fatty degeneration of hepatocytes with dilatation of the blood vessels (Figure 6c). Group 3 B (diabetic non-treated) displayed intensive necrosis and fatty degeneration of hepatocytes with thickening and hyperplasia of blood vessels wall (Figure 6d). Group 4 A (prediabetic treated) detected a mild degree of fatty degeneration and dilatation of the blood sinusoids (Figure 6e). The liver of group 4 B (diabetic treated) revealed slight fatty degeneration of hepatocytes with slight dilatation of blood sinusoids (Figure 5f).

### Pathological Findings of the Kidney in Control and Diabetic Rats

**Figure 7:**
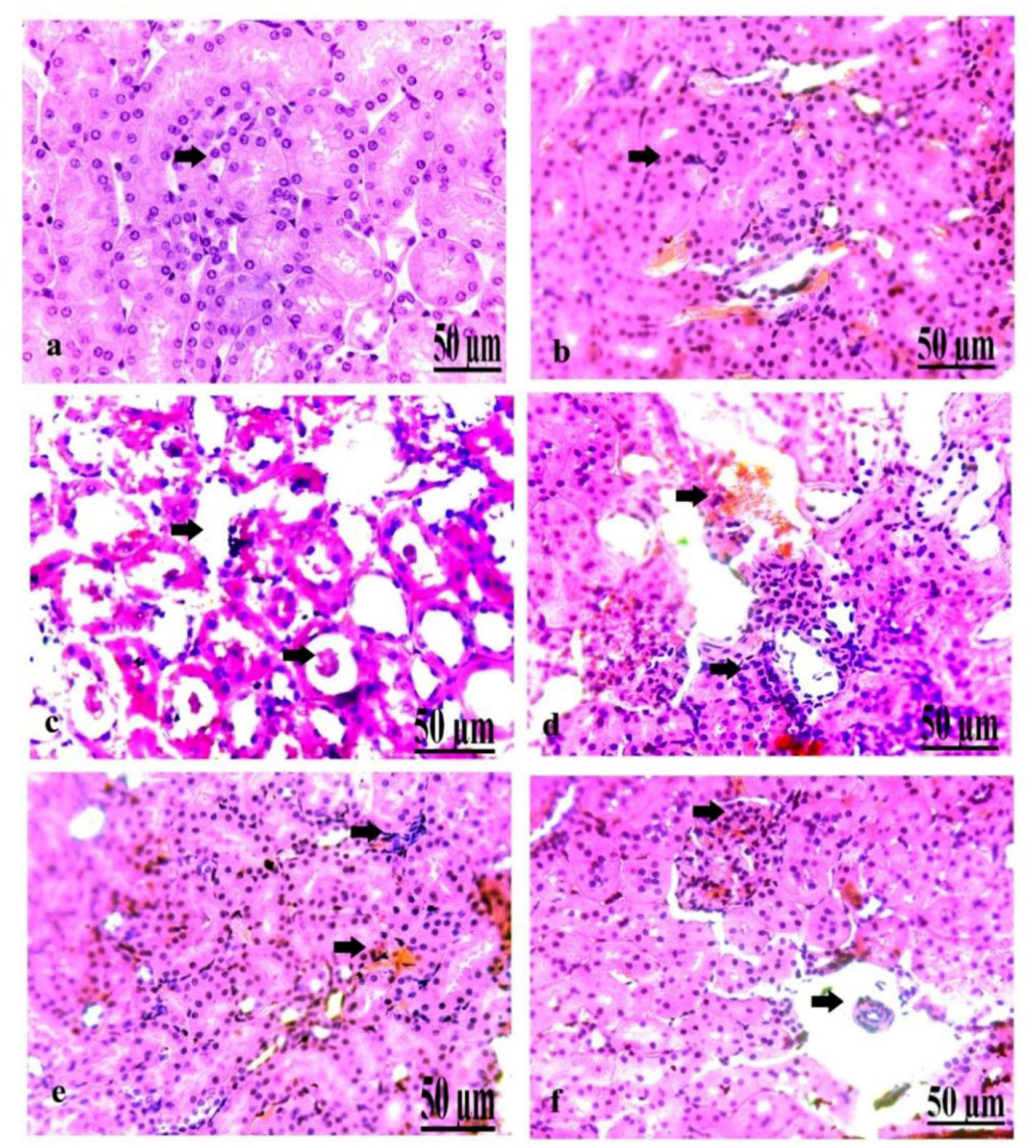
**(a–f)** Photomicrograph of the kidney of the control group showing normal nephrons **(a)**. Photomicrograph of the kidney of the oxytocin group showed mild hemorrhage and congestion of the blood vessels, with some renal necrotic changes **(b)**. Photomicrograph of the kidney of group 3 A (prediabetic non-treated) showing slight necrosis of renal tissues with congestion and dilatation of the blood vessels **(c)**. Photomicrograph of the kidney of group 3 B (diabetic non-treated) showed moderate congestion and dilatation of the interstitial blood vessels and glomeruli, besides slight necrosis of the renal tubules **(d)**. Photomicrograph of the kidney of group 4 A (prediabetic treated) showed a moderate degree of congestion of the blood vessels, with some necrosis of the renal tissues **(e)**. Photomicrograph of the kidney of group 4 B (diabetic treated) showed slight congestion and hypercellularity of the glomeruli with the degree of necrosis of the renal tubules **(f)** (H&E., bar = 50 μm).

On a pathological basis, we analyzed the kidney (Figure 7). The microscopic findings of the control group showed normal nephrons characterized by normal renal tubules and glomeruli (Figure 7a). The kidney of the oxytocin group suffered from mild hemorrhage and congestion of the blood vessels, with some renal necrotic changes (Figure 7b). Group 3 A (prediabetic non-treated) displayed slight necrosis of renal tubules with congestion and dilatation of the blood vessels (Figure 7c). The kidney of group 3 B (diabetic non-treated) showed moderate congestion and dilatation of the interstitial blood vessels and glomeruli, and slight necrosis of the renal tubules (Figure 7d). Group 4 A (prediabetic treated) detected a moderate degree of congestion of the blood vessels, with some necrosis of the renal tissues (Figure 7e). Group 4 B (diabetic-treated) showed slight congestion and hypercellularity of the glomeruli with the degree of necrosis of the renal tubules (Figure 7f).

### Correlation Between the Biochemical Parameters, *PEPCK*, and the Behavioral Activities With the *FBN1* Expression in Pre-diabetic and Diabetic Wistar Rats

As shown in (Table 5), motor activities such as locomotion, general activities, rearing, stereotyped behaviors, and fast movement were negatively correlated (−0.61, −0.55, −0.32, −0.52, and −0.42) with *FBN1* gene expression in prediabetic rats, while it was (−0.64, −0.57, −0.39, −0.56, and −0.47) in diabetic ones. Moreover, the body weight (+0.41), glucose level (+0.54), insulin (+0.31), HOMA-IR (+0.22), ALT (+0.53), AST (+0.44), *PEPCK mRNA* expression (+0.33), TC (+0.54), TAG (+0.66), LDL-c (+0.55), VLDL-c (+0.63), and creatinine (+0.33) were positively correlated with *FBN1* in prediabetic rats, while it was (+0.49, +0.66, +0.35, +0.28, +0.67, +0.48, +0.37, +0.61, +0.67, +0.57, +0.59, and +0.38) in diabetic ones. However, insulin (−0.35) in diabetic rats and albumin (−0.36, −0.41 in prediabetic and diabetic, respectively), and *OLR1612* expression (−0.32, −0.35 in prediabetic and diabetic, respectively), were negatively correlated with the *FBN1* gene. HDL-c has no correlations in all groups.

**Table 5:** The correlation coefficients between biochemical parameters, *PEPCK*, and the behavioral activities with *FBN1* gene expression in prediabetic and diabetic Wistar rats.

***FBN1* gene**	**Pre-diabetic** **(normal)**	**Pre-diabetic + OX** **(normal)**	**Diabetic** **(HFHSD)**	**Diabetic + OX** **(HFHSD)**
**Biochemical parameters and** ***PEPCK*** **gene**				
Body weight (gm)	+0.41^a^	−0.33^a^	+0.49^a^	−0.37^a^
Glucose (mg/dl)	+0.54^b^	−0.51^b^	+0.66^b^	−0.56^b^
Insulin (mIU/L)	+0.31^a^	−0.41^a^	−0.35^a^	+0.46^a^
HOMA-IR	+0.22^a^	−0.35^a^	−0.28^a^	+0.37^a^
ALT (U/L)	+0.53^b^	−0.55	+0.67^b^	−0.62^b^
AST (U/L)	+0.44^a^	−0.33^a^	+0.48^a^	−0.42^a^
Hepatic *PEPCK* enzyme gene	+0.33^a^	−0.42^a^	+0.37^a^	−0.45^a^
HDL-c (mg/dl)	0.089	0.011	0.09	0.022
LDL-c (mg/dl)	+0.55^b^	−0.64^b^	+0.57^b^	−0.67^b^
VLDL-c (mg/dl)	+0.63^b^	−0.52^b^	+0.59^b^	−0.51^b^
TC (mg/dl)	+0.54^b^	−0.53^b^	+0.61^b^	−0.51^b^
TAG (mg/dl)	+0.66^b^	−0.55^b^	+0.67^b^	−0.63^b^
Creatinine (mg/dl)	+0.33^a^	−0.49^a^	+0.38^a^	−0.41^a^
Albumin (g/dl)	−0.36^a^	+0.50^a^	−0.41^a^	+0.44^a^
**Motor activities/seconds**				
Locomotion	−0.61^b^	+0.53^b^	−0.64^b^	+0.58^b^
General activity	−0.55^b^	+0.64^b^	−0.57^b^	+0.67^b^
Distance	−0.64^b^	+0.58^b^	−0.66^b^	+0.59^b^
Stereotypic behavior	−0.52^b^	+0.67^b^	−0.56^b^	+0.68^b^
Resting time	+0.64^b^	−0.51^b^	+0.63^b^	−0.54^b^
Slow movement	+0.50^a^	−0.32^a^	+0.44^a^	−0.35^a^
Number of rearing	−0.32^a^	+0.43^a^	−0.39^a^	+0.46^a^
Fast movement	−0.42^a^	+0.35^a^	−0.47^a^	+0.37^a^

*Each value represented Spearman's rho correlation coefficient and Pearson Correlation Coefficients. ALT, alanine transaminase; AST, aspartate transaminase; FBN1, Fibrillin-1 gene; HDL-c, high-density lipoprotein cholesterol; HFHSD, high-fat-high-sugar-diet; HOMA-IR, homeostatic model assessment of insulin resistance; LDL-c, low-density lipoprotein cholesterol; PEPCK, phosphoenolpyruvate caboxykinase; TAG, triacylglycerol; TC, total cholesterol; VLDL-c, very low-density lipoprotein cholesterol; and OX; oxytocin*.

a *(r) = 0.3–0.5, weak to moderate correlation to FBN1 gene*.

b *(r) = 0.5–0.7, moderate to strong correlation to FBN1 gene*.

*^c^ (r) = 0.7, strong correlation to FBN1 gene*.

## Discussion

Diabetic-associated obesity is a major disease that leads to death (2). Thus, it is essential to find a biomarker for diabetes prognosis and targeted intervention for diagnostic therapy. Asprosin is a hormone made up of proteins and produced from WAT, and its levels are abnormally high in diabetic individuals. However, information about the role of this hormone in T2DM remains unclear. In response, the present study evaluated the role of asprosin for DM progress. As we understand it, this is a novel study focused on the relation between diet-induced diabetes and obesity to assess the level of genetic expression of *FBN1* in the brain and enzymatic activity of *PEPCK* in the liver and to demonstrate the influence of oxytocin on some biochemical parameters to combat diabetes.

The obtained results showed increasing significantly in body weight in prediabetic and diabetic (*P* < 0.01, *P* < 0.001), and an increase in glucose release due to diet-induced obesity, along with experimental duration in comparison to the control rats. As a result, we noticed that control rats normally suffer from abundant weight loss starting from the 10th week onwards in all groups. However, it was reported that diabetic rats provoked by streptozotocin suffered from weight loss and they referred to this to a loss in TAG stores and muscle mass, or outlined that it was probably because of the storage of insulin that led to increased amino acid anabolism by tissue synthesis, and enhanced lipolysis and protein breakdown in adipose tissues (30). As a result, in this investigation, we opted to use diet-induced diabetes and obesity rather than using streptozotocin to avoid conflicting data and to monitor the normal metabolism state correctly. Furthermore, subcutaneous oxytocin injection reduced food intake in rodents and rhesus monkeys, which was linked to higher energy expenditure (31). In humans, oxytocin has been shown to have an immediate inhibitory effect on food consumption (32).

Oxytocin is a hormone most generally associated with labor and lactation. It has an extensive range of physiological and pathological functions, which makes oxytocin and its receptor capacity have potential as drug remedies (33). Oxytocin may additionally have fine metabolic outcomes. This is based totally on the alternate in glucose metabolism, lipid profile, and insulin sensitivity. It may regulate glucose uptake and insulin sensitivity through direct and indirect consequences. It could additionally purpose regenerative changes in diabetic Islet cells of the pancreas. The activation of the oxytocin receptor pathway by means of infusion of oxytocin, oxytocin analogs, or oxytocin agonists may additionally constitute a promising approach for the control of obesity and associated metabolic illnesses in addition to diabetes and its complications. Furthermore, oxytocin improves insulin sensitivity by (1) lowering glucotoxicity and lipotoxicity, (2) regulating cytokines like leptin and adiponectin, and (3) oxytocin decreased fat mass, ensuing in a reduction in the leptin stage (31).

It is well-known that oxytocin has a useful impact on glucose homeostasis. For instance, 1-month medication of oxytocin has decreased plasma glucose concentrations in monkeys (31). Moreover, in adults, a single dose of oxytocin proved beneficial in reducing the peak circulating glucose resulting from breakfast ingestion, and significantly improved the sensitivity of insulin, such as decreased insulin concentrations during fasting conditions and HOMA-IR (32). The obtained results indicated that oxytocin enhanced insulin secretion in animals (34). In young rats' muscle cells, oxytocin triggers glucose uptake (13, 35). In rats, the occurrence of oxytocin receptors in the Langerhans islets helps in the regulation of insulin and glucagon absorption. In this context, targeting the oxytocin protocol is advanced as an effective therapeutic strategy for metabolic disorders (35).

Oxytocin has useful impacts on glucose homeostasis. For instance, 1-month medication of oxytocin decreased plasma glucose concentrations in monkeys (31). Moreover, in adults, a single dose of oxytocin proved beneficial in reducing the peak circulating glucose resulting from the breakfast ingestion, and significantly improved the sensitivity of insulin, such as decreased insulin concentrations during fasting conditions and HOMA-IR (32). Our findings are in line with earlier findings, indicating that oxytocin enhanced insulin secretion in animals (34). In young rats' muscle cells, oxytocin triggers glucose uptake (13, 35). In rats, the occurrence of oxytocin receptors in the Langerhans islets helps in the regulation of insulin and glucagon absorption. In this context, targeting the oxytocin protocol is advanced as an effective therapeutic strategy for metabolic disorders (35).

An obese-diabetic patient suffered from lower oxytocin than normal levels in the blood, meaning there is a negative relationship between the release of oxytocin and glucose. However, its involvement in the metabolism and homeostasis of glucose is unknown, particularly in humans (13). This is because obesity is considered one of the major features of diabetes, and is already seen as an epidemic concern and a public health problem worldwide. The hypothalamus responds to neurotransmitters secreted from the gastrointestinal tract, making the central nervous system a target organ in the control of adiposity (36). Thus, appetite is a complex neurometabolic feeling that controls the physiological processes in reaction to fasting hormones. We reported that fasting can increase plasma levels of asprosin and induce the hepatic release of glucose, but eating blocks its production (37). Therefore, oxytocin regulates metabolism by altering eating habits and energy expenditure. Because of the great benefits of using oxytocin for the treatment of diabetes, we decided to treat the control, pre-diabetic, and diabetes groups with *intra*-peritoneal injection of oxytocin as a faster and safer route than other routes of medicine administration as it does not interfere with the rat's physical metabolism.

Oxytocin has been shown to suppress appetite, induce weight loss, and improve glycemic control and lipid metabolism in several species, including humans, monkeys, and rodents. However, oxytocin's short half-life in circulation and lack of receptor selectivity limit its application and efficacy. However, the oxytocin peptide analog (OXT^Gly^) is potent and selective for the oxytocin receptor. Therefore, selective activation of the oxytocin receptor pathway results in both acute and chronic metabolic benefits, whereas potential activation of vasopressin receptors by non-selective oxytocin analogs causes physiological stress that contributes to additional weight loss (38).

Oxytocin prevents marked deterioration in pancreatic ß-cell function with subsequent marked improvement in insulin secretion in comparison to untreated diabetes. This is in line with the findings of other researchers, who stated that oxytocin protected ß-cells by acting as an anti-inflammatory and antioxidant, as well as protecting against oxidative stress caused by diet-induced obesity (24, 39). Similarly, oxytocin contributed to the control of blood glucose levels as it promoted glucose uptake in rats and its link to insulin secretion is because of the occurrence of receptors of insulin in Langerhans islets (12). In addition, oxytocin increased ß-cell responsiveness and glucose tolerance in healthy people (9). Furthermore, glucose tolerance improves in response to oxytocin in diet-induced obesity and diabetes through preventing ß-cell death from obesity state (19).

The influence of oxytocin on insulin secretion is regulated centrally through vagal cholinergic neurons, which innervate ß-cells, and peripherally through inducing the activities of phosphoinositide and protein *kinase C* in ß-cells (13). Our results confirmed that oxytocin improved ß-cell function as evidenced by increased serum insulin in pre-diabetic and diabetic groups, and therefore slightly closed to the values of the control group. Our result was supported by another study stating that pancreatic ß-cell function was measured by serum *C*-peptide and insulin (40). Interestingly, it was found that adipocytes were observed to be smaller in the adipose tissue of rats given oxytocin compared to controls. Thus, the activity of proliferator- receptor-y for modulating the development of adipocytes supports this theory (41). This inconsistency is probably because of the difference in the research condition as our study evaluated the influence of oxytocin on diabetic rats.

Treated diabetic rats with oxytocin had a considerable drop in blood glucose levels, which was used as a reliable index of diabetic control (42). This was also supported by oxytocin's decreased blood glucose level in diabetes through its capacity to increase peripheral glucose uptake through an insulin-like signaling pathway (13). This was further supported by the oxytocin stimulated glucose oxidation and increased lipolysis and fatty acids ß-oxidation via its direct effect on adipocytes. Meanwhile, our result was in line with those authors who stated that the production of pancreatic ß-cells in diabetic rats is caused by the lower glucose levels in the treated diabetics (43). However, our results disagreed with those who claimed that treatment of diabetic rats involved increases in their body weights, which they attributed to improved insulin secretion and action (42) because in the present study we could not ensure weight gain since the weight of the control rat dramatically decreased from the 10th week and the obese rat became partially steady until the end of the experiment at 16th week. Oxytocin-treated diabetic rats showed similarities in body mass with those of healthy rats (44).

Decreasing the atherogenic index after treatment of diabetes was perfect and they referred to this effect as a decrease in levels of LDL-c (bad cholesterol). It is essential that it is low to avoid transporting cholesterol to the liver from peripheral cells and is a cardioprotective lipid (45). HDL-c also protects membranes and lipid metabolism from oxidative stress by reverse cholesterol transporting cholesterol from peripheral tissues to the liver (46).

We also assessed liver function changes in the pre-diabetic group after oxytocin treatment and comparing the results with those of diabetic rats. Findings showed a significant reduction in blood levels of ALT and AST (*P* < 0.01, *P* < 0.001) but significant increases (*P* < 0.05 each) in the blood levels of albumin were found. These results confirmed that oxytocin had protective effects on liver functions in cases of HFHSD-induced diabetes of its early stage.

Our results supported the hypothesis of the study and evidenced the particularly protective effect of oxytocin to inhibit both, neutrophil migration, and the release of pro-inflammatory cytokines (47). Similarly, there were significant decreases in serum levels of hepatic enzymes ALT (in prediabetic and diabetic, *P* < 0.05 each), AST (in prediabetic and diabetic, *P* < 0.01 each), but the albumin levels in the blood had increased significantly (in prediabetic and diabetic, *P* < 0.05 each) with the treatment of oxytocin-induced diabetes, which agreed with a previous report (30). Therefore, oxytocin improved the hepatic function of diabetic cases. The results of this study in the post-oxytocin group also demonstrated a significant reduction in serum glucose as well as lipid profile, but a considerable increase in the serum insulin levels, compared to the untreated group; as we mentioned their significance levels above.

The asprosin-related gene is thought to play a role in kidney dysfunction which was evidenced by increasing the serum creatinine conc in prediabetic/diabetic Wistar rats to promote inflammatory responses via the TLR4/JNK-mediated signal pathway. However, oxytocin modulated inflammatory processes, by stimulating phagocyte migration through affecting levels of growth hormone (48, 49), which here ensures the biological role of oxytocin. The mechanisms underlying oxytocin's effect on glucose homeostasis have yet to be discovered. Thus, glucose sensors have been discovered in oxytocin neurons in the hypothalamic supraoptic nucleus (50). However, it is unclear if oxytocin's impact in glucose homeostasis is dominated by central or peripheral pathways (34, 51), not least because oxytocin may activate its own centers via a releasing mechanism after peripheral injection.

Oxytocin has also been demonstrated to influence the control of cytokines like adiponectin, as well as lowering hyperglycemia and lipotoxicity (52). To establish the proportional integrations of various metabolic mechanisms involved with oxytocin's glucoregulatory actions, more research on isolated pancreatic islets is needed. Hormonal variables can mediate the glucose-regulating impact of oxytocin as a result of the interplay between additional endocrine signaling systems and oxytocin (35). The initial increase in insulin concentrations after oxytocin injection, followed by a little drop in peak insulin responses 50 min later, is consistent with reports of oxytocin stimulating insulin production in animals and/or humans (19, 34, 53). It is believed that the release of insulin in the first phase is particularly relevant to glucose tolerance, because abnormalities in this route may signal the onset of diabetes changes (53). However, in healthy patients of normal weight, it was reported that the reduction of the resulting peak glucose responses was independent from the initial insulin release. In healthy people, the effects of oxytocin on glucose tolerance and ß-cell responsiveness have been well-investigated.

The decreasing and increasing expressions of asprosin after stress or even exercise, give rise to questions about the mechanism pathway of asprosin (38). Specifically, asprosin research focused on glucose and lipid metabolism (14), revealing mechanisms of the metabolic dysfunction of glucose and lipid metabolic dysfunction. However, it is unclear whether asprosin is caused by a protective feedback mechanism or by metabolic abnormalities. In parallel, multiple studies have validated the link between glucose and lipid metabolism. Herein, we are interested to identify the metabolic role of asprosin *in vivo*. In our results, the biochemical parameters indicated an increase of TAG, LDL-c, VLDL-c, and creatinine in diabetics compared to control rats. Furthermore, in diabetic rats fed HFHSD, there was an abnormal lipid profile in the form of elevated TAG, LDL-c, VLDL-c, and TC, but decreased HDL-c and they declared that LDL-c proteins were more vulnerable to oxidation and were considered to be pro-atherogenic.

On assessing liver function changes in obese-diabetic treated rats, increases in the serum levels of ALT and AST were found. These results confirmed the occurrence of deteriorated liver functions and it was evidenced by the presence of intracellular enzymes, high levels of AST and ALT used to assess the hepatocellular injury, in the obese diabetic rat model. Also, it was stated that the presence of elevation in serum ALT in diabetic patients was associated with poorer glycemic control and raised TAG (24). Therefore, the dysfunctions of the liver in obese-treated rats indicated the significant increases in serum levels of hepatic enzymes. However, a significant decrease in serum albumin levels indicated the incidence of hepatocellular damage. This phenomenon might be due to the oxidative stress that occurred secondary to hyperglycemia, which decreased antioxidant levels and enhanced free radicals (54). Herein, the albumin level was decreased in the diabetic rat model, which confirmed the occurrence of oxidative stress. While the results of this study in the pre-diabetic group treated with oxytocin recorded a reduction in serum glucose of significance (*P* < 0.05), TAG (*P* < 0.001), LDL-c, and VLDL-c (*P* < 0.01 each), and an abundant increase in serum insulin levels and HDL-c (*P* < 0.05) when compared to the prediabetic group.

In this study, correlations in the obese-treated group between *FBN1* genetic expression and different studied parameters declared positive associations with final body weight (weak correlation), glucose (moderate correlation), insulin in prediabetics (weak correlation), hepatic enzymes (ALT “moderate correlation,” AST “weak correlation”), (TC, TAG, LDL-c, and VLDL-c; moderate correlation each), and creatinine (weak correlation). We also found negative associations with serum insulin in diabetes and albumin (weak correlation each), but HDL-c was not correlated with biochemical parameters. In addition, there is a moderate positive correlation with motor activities like locomotion, general activities, rearing, and stereotyped behaviors. Therefore, these results indicate that changes in asprosin level affected glucose homeostasis, lipid profile, hepatic and renal functions, and subsequently the pathogenesis and complications of DM (14, 30).

Literature on the relation between the *FBN1* gene and behavioral disorders is limited. On the other hand, anorexia nervosa has been described as a psychiatric disease marked by self-inflicted weight loss and appetite suppression. Interoceptive awareness includes both the observation and attention to the underlying urges as well as the sensation of the physiological state of the body, such as temperature, pain, hunger, and vasomotor activity. Therefore, the authors of this study believe the aberrant interaction between asprosin and insulin is the responsible link for how insulin plays a key role in anorexia nervosa symptoms and behavioral manifestations (55). The depressive-like behavior of diabetic-rat might result from increasing circulatory asprosin hormone and/or *FBN1* expression, and therefore oxytocin medication significantly improved these behavioral patterns, such as the locomotion, general activity, distance, stereotypic activity, number of rearing, and movement (*P* < 0.01, *P* < 0.001, *P* < 0.01, *P* < 0.01, *P* < 0.01, and *P* < 0.05, respectively), in a diabetic rat model.

Diabetic rats had a higher level of asprosin in their blood when compared to the prediabetic and control rats. Because plasma asprosin level was altered by pancreatic ß-cell function, this could have happened as a result of the progressive increase in blood glucose level and increase in serum insulin level (56). In our previous review, we found that an asprosin-specific monoclonal antibody diminished circulating asprosin and enhanced insulin sensitivity in rats with insulin resistance (38). Our results revealed that *FBN1*-*mRNA* expression is significantly increased in prediabetic and diabetic Wistar rats (*P* < 0.01 each) due to HFHSD compared with those in healthy controls. As previously suggested, the increase in *FBN1* expression may be linked to the increase in body weights and changes in serum glucose and insulin in obese-treated rats (3). This confirmed that *FBN1* genetic expression fluctuated depending on the alterations of glucose and insulin levels in the blood and subsequently, glucose served as a helper of circulating asprosin levels. They also declared that an increased asprosin level was accompanied by increased insulin secretion glucose metabolism (57). This means that serum asprosin concentrations were abundantly increased in diabetic progress. Meanwhile, *FBN1* expression was considerably reduced in both diabetic and healthy rats (*P* < 0.01, *P* < 0.001, respectively), in post-oxytocin groups in comparison to the untreated ones. This may be due to the changes that occurred in serum levels of glucose and insulin, which confirmed that asprosin had a role in the pathogenesis of obese-induced diabetes.

The obtained results are in parallel to previous results (6), which indicated that the concentration of asprosin hormone was shown to be significantly greater in diabetic individuals, implying that this hormone could be a risk factor for T2DM pathogenesis. It means that the circulatory asprosin is gradually increased in new-onset T2DM patients and severe diabetic ones. Hence, *FBN1* genetic expression indicated the evidence of asprosin level/expression. It, therefore, could be considered a new biomarker to make the best prognosis of diabetes.

The *PEPCK* is the main enzyme that regulates glycogenolysis and gluconeogenesis. Enhanced *PEPCK* gene expression in the hepatic tissues was confirmed in the diabetes model and linked to increased glycogenolysis, according to this study. Insulin inhibits gluconeogenesis and down-regulated the *mRNA* encoded *PEPCK* (58). As a result, in diabetic rats, the PEPCK expression increased, whereas it decreased in oxytocin therapy groups. The results of this investigation are similar to those of a prior study (59, 60), which reported insulin-regulated *PEPCK mRNA* expression levels in diabetic rats. Insulin regulates *PEPCK* activity at the transcriptional level. Glucose synthesis was suppressed by *PEPCK* gene transcription when insulin levels were high. Herein, because circulating insulin levels were lower in oxytocin-treated rats than controls, insulin levels could be affected by changes in hepatic glucose-regulating enzymes (61). In diabetic rats, hepatic glycogenolysis controlled plasma glucose levels, suggesting that *glycogen phosphorylase* inhibitors may be effective in the treatment of diabetes (62).

The *PEPCK-C* catalyzes an irreversible step of gluconeogenesis, the process whereby glucose is synthesized. The enzyme has therefore been thought to be essential in glucose homeostasis, as evidenced by laboratory mice that contracted diabetes mellitus type 2 as a result of the overexpression of *PEPCK-C* (63). The role that *PEPCK-C* plays in gluconeogenesis may be mediated by the citric acid cycle, the activity of which was found to be directly related to *PEPCK-C* abundance (64). The same author reported that *PEPCK-C* levels alone were not highly correlated with gluconeogenesis in the mouse liver, as previous studies have suggested. While the mouse liver almost exclusively expresses *PEPCK*, humans equally present a mitochondrial isozyme (*PEPCK-M*). The *PEPCK-*M has gluconeogenic potential *per se* (65).

Insulin-resistant rats have pathologically elevated plasma oxytocin levels. A decrease in its function due to immunological or genetic factors leads to stronger glucose, which has the effect of reducing insulin as a result of glucose deficiency in the liver (14). From the view of the cross-section, the diverse histological alterations detected in variant organs involving pancreas, liver, and kidney after induction of diabetes and obesity comprised necrosis with destructive damage, besides congestion of the blood vessels. This eventually resulted from distinct oxidative stress attributed to chronic persistent hyperglycemia as well as hyperlipidemia, which impaired the antioxidant defense system, and thus damaged cell membranes resulting in a release of reactive oxygen species (ROS) (66). Furthermore, excessive ROS is linked to decreased insulin production and release, as well as a variety of organs including the liver, kidney, and the hematopoietic system (67). We confirmed an elevation in the hepatic enzymes like ALT, AST of diabetic rats supplemented on HFHSD, which led to fatty degeneration of hepatocytes. This is dues to leakage from enzyme cells cytosols into the blood circulation, a detrimental effect of obesity negatively affecting liver integrity that leads to hepatic injury and extensive damage through the release of oxidative free radicals (68).

In addition to the previous results, the elevated level of serum creatinine due to diabetes correlated with extensive damage and necrosis observed in the kidney under influence of the damaged pancreatic cells (69). Contradictory, oxytocin could to some extent restore and correct the destructive effect induced in tissues by diabetes. Oxytocin could avoid β-cells apoptosis, damage with the maintenance of its neogenesis (70). The histological view indicates an improvement in biochemical findings. Oxytocin has been shown to have antioxidant and anti-inflammatory properties (71). It has an anti-diabetic effect by lowering oxidative damage and inflammation in β-cells. Meanwhile, variable antidiabetic drugs act on peripheral tissues and do not directly affect pancreatic islets (72). However, the ß-cell inflammatory process occurred as a result of dyslipidemia, hyperglycemia, increased circulating pro-inflammatory adipocytokines, and toxic molecules such as ROS, which acted significantly in islet cell death by inducing *DNA* damage (73). Moreover, it was reported that *C-*peptide was formed in ß-cell of the pancreas by cleavage of pro-insulin to an equal amount of *C-*peptide and insulin, which indicates that endogenous insulin production can be measured using *C*-peptide (40). These results confirmed that exogenous oxytocin treatment after induction of diabetes by HFHSD improved manifestations of DM. Therefore, a typical diabetic profile was achieved by combining all biochemical, molecular, and behavioral investigations with asprosin hormones appearing to be a master key in targeting a possible biomarker in T2DM.

In this study, correlations in the obese-treated group between *FBN1* genetic expression and different studied parameters revealed positive associations with final body weight (weak correlation), glucose (moderate correlation), insulin in prediabetics (weak correlation), hepatic enzymes (ALT “moderate correlation,” AST “weak correlation”), (TC, TAG, LDL-c, and VLDL-c; moderate correlation each), and creatinine (weak correlation. There were also positive correlations with serum insulin in diabetes and albumin (weak correlation each), but HDL-c was not correlated with biochemical parameters. In addition, there is a moderate positive correlation with motor activities like locomotion, general activities, rearing, and stereotyped behaviors. Therefore, these results declared that asprosin level changes affected glucose homeostasis, lipid profile, hepatic and renal functions, and subsequently the pathogenesis and complications of DM (14, 30). However, further studies have revealed that a selective oxytocin peptide analog should be used for the long action. The biochemical influences of asprosin on different body organs under different metabolic syndrome should also be performed. In addition, studies should analyze the serum asprosin hormone level in diabetic patients to confirm the effect of the asprosin-related gene (*FBN1*).

## Conclusions

Diabetic-associated obesity is an increasingly common disease that takes a deadly toll on human health, leading to several complications. A biomarker of diabetes is needed to predict its prognosis. Our results indicated that the asprosin related gene (*FBN1*) was closely associated with body weight, glucose metabolism, and insulin secretion in all groups including healthy and diseased rats. Oxytocin was used as antidiabetic and antiobesity effects to suppress appetite and improve glycemic and lipid profiles. The *FBN1* gene was significantly correlated with a profile of adiposity, glucose, and insulin metabolism, even after adjusting for age. After the subgroup analyses, we noticed that the average *FBN1* expression was higher in overweight/obese subgroups. Asprosin levels were significantly decreased in cases of post-oxytocin treatment in comparison with the control Wistar rats and with the group fed HFHSD. On the other hand, oxytocin improved motor activities in diabetic rats and after oxytocin therapy. It modulated the pathological lesions of internal organs in diabetic rats. The *FBN1* gene was significantly correlated with a profile of adiposity, glucose, and insulin metabolism, even after adjusting for age in the obesity-induced T2DM. Therefore, the genetic expression of *FBN1* and *PEPCK* enzyme activity in diabetic rats and after oxytocin therapy indicated the possibility that asprosin could be a biomarker of diabetes progression.

## Data Availability Statement

The data presented in the study are included in the article/supplementary material, further inquiries can be directed to the corresponding author/s.

## Ethics Statement

The animal study was reviewed and approved by Institutional Review Board Statement: Animal Experimental Guidelines were followed, and the Animal Care and Use Committee of the Vet-Med Faculty, Zagazig University, Egypt, authorized protocol number ZU-IACUC/2/F/21/2019.

## Author Contributions

AE, KE-D, HE-B, EM, MY, and IR jointly developed the hypothesis and carried out the main study. OS, ZA-A, EM, SA, SC, and SF were all involved in the experimental procedures and analyses. All of the authors participated in the experimental analysis and they helped with the manuscript's rewriting. All authors read and approved the final manuscript.

## Funding

The authors would like to express their gratitude to the King Saud University (Riyadh, Saudi Arabia) for the funding of this research through Researchers Supporting Project number (RSP-2021-241). This work was also supported by Grant No. 2505 from the Science and Technology Development Fund (STDF), Egypt.

## Conflict of Interest

The authors declare that the research was conducted in the absence of any commercial or financial relationships that could be construed as a potential conflict of interest.

## Publisher's Note

All claims expressed in this article are solely those of the authors and do not necessarily represent those of their affiliated organizations, or those of the publisher, the editors and the reviewers. Any product that may be evaluated in this article, or claim that may be made by its manufacturer, is not guaranteed or endorsed by the publisher.

## References

[1] Ogurtsova K da Rocha Fernandes JD Huang Y Linnenkamp U Guariguata L Cho NH. Diabetes atlas: global estimates for the prevalence of diabetes for 2015 and 2040. Diabetes Res Clin Pract. (2017) 128:40–50. 10.1016/j.diabres.2017.03.024 28437734

[2] Conte C Fabbrini E Kars M Mittendorfer B Patterson BW Klein S. Multiorgan insulin sensitivity in lean and obese subjects. Diabetes Care. (2012) 35:1316. 10.2337/dc11-1951 22474039 PMC3357234

[3] Muthu ML Reinhardt DP. *Fibrillin-1* and *Fibrillin-1*-Derived asprosin in adipose tissue function and metabolic disorders. J Cell Commun Signal. (2020) 142:159–73. 10.1007/s12079-020-00566-3 32279186 PMC7272526

[4] Feng B Xu P He Y. Novel targets in glucose homeostasis and obesity—lesson from rare mutations. Curr Diabetes Rep. (2020) 20:1–10. 10.1007/s11892-020-01351-7 33128381

[5] Liu Y Long A Chen L Jia L Wang Y. The asprosin - OLFR734 module regulates appetitive behaviors. Cell Discov. (2020) 6:1–3. 10.1038/s41421-020-0152-4 32337066 PMC7154029

[6] Zhang L Chen C Zhou N Fu Y Cheng X. Circulating asprosin concentrations are increased in type 2 diabetes mellitus and independently associated with fasting glucose and triglyceride. Clin Chim Acta. (2019) 489:183–8. 10.1016/j.cca.2017.10.034 29104036

[7] Ceylan HI Saygin Ö Türkcü ÜÖ. Assessment of acute aerobic exercise in the morning versus evening on asprosin, spexin, lipocalin-2, and insulin level in overweight/obese versus normal weight adult men. Chronobiol Int. (2020) 37:1252–68. 10.1080/07420528.2020.1792482 32741294

[8] Rezk MY Elkattawy HA Fouad RA. Plasma asprosin levels changes in pregnant and non-pregnant rats with and without gestational diabetes. Int J Med Res Heal Sci. (2020) 9:54–63. Available online at: https://www.ijmrhs.com/abstract/plasma-asprosin-levels-changes-in-pregnant-and-nonpregnant-rats-with-and-without-gestational-diabetes-44844.html

[9] Klement J Ott V Rapp K Brede S Piccinini F Cobelli C. Oxytocin improves β-cell responsivity and glucose tolerance in healthy men. Diabetes. (2017) 66:264–71. 10.2337/db16-0569 27554476

[10] Aulinas A Plessow F Asanza E Silva L Marengi DA Fan W. Low plasma oxytocin levels and increased psychopathology in hypopituitary men with diabetes insipidus. J Clin Endocrinol Metab. (2019) 104:3181. 10.1210/jc.2018-02608 30882859 PMC6570634

[11] Akour A Kasabri V Bulatova N Muhaissen SAl Naffa R Fahmawi H. Association of oxytocin with glucose intolerance and inflammation biomarkers in metabolic syndrome patients with and without prediabetes. Rev Diabet Stud. (2017) 14:364–71. 10.1900/RDS.2017.14.364 29590229 PMC6230448

[12] Suzuki M Honda Y Li MZ Masuko S Murata Y. The localization of oxytocin receptors in the islets of langerhans in the rat pancreas. Regul Pept. (2013) 183:42–5. 10.1016/j.regpep.2013.03.019 23500836

[13] Weingarten MFJ Scholz M Wohland T Horn K Stumvoll M Kovacs P. Circulating oxytocin is genetically determined and associated with obesity and impaired glucose tolerance. J Clin Endocrinol Metab. (2019) 104:5621–32. 10.1210/jc.2019-00643 31361301

[14] Ramnanan CJ Edgerton DS Rivera N rimia-Dominguez J Farmer B Neal DW. Molecular characterzation of insulin-mediated suppression of hepatic glucose production *in vivo*. Diabetes. (2010) 59:1302–11. 10.2337/db09-1625 20185816 PMC2874690

[15] Yabaluri N Bashyam MD. Hormonal regulation of gluconeogenic gene transcription in the liver. J Biosci. (2010) 35:473–84. 10.1007/s12038-010-0052-0 20826956

[16] Abd-Allah AB Megahed AAY Gomaa RS Hussein SFE. Effect of moderate intensity exercise on serum visfatin level in male rat model of obesity. AAMJ. (2013) 11. Available online at: http://www.aamj.eg.net/inner/jarticle.aspx?aid=2045

[17] Zhang H Wu C Chen Q Chen X Xu Z Wu J Cai D. Treatment of obesity and diabetes using oxytocin or analogs in patients and mouse models. PLoS ONE. (2013) 8:e61477. 10.1371/journal.pone.0061477 23700406 PMC3658979

[18] El-naggar A El-Dawy K. Role of micro RNAs and its associated genes in prediction of obesity and diabetes mellitus (type II). Zag Vet J. (2019) 47:57–67. 10.21608/zvjz.2019.6135.1010

[19] El-Gayar KE Ibrahim MA Mohamed SH Zakaria Z Alhameed ASA. Application of extracted peroxidase enzyme from turnip roots (BRASSICA NAPUS) in clinical diagnostic kit. Intl J Curr Res Rev. (2012) 4:1–2. Available online at: https://www.ijcrr.com/uploads/1580_pdf.pdf

[20] Samuel I Arthur N Jude E Henrietta C. Antihyperglycaemic efficacy of *Cnidoscolus Aconitifolius* compared with glibenclamide in alloxan-induced diabetic wistar rats. Intl Res J Med Sci. (2014) 2:1–4. Available online at: www.isca.in; www.isca.me

[21] Naiemian S Naeemipour M Zarei M Lari Najafi M Gohari A Behroozikhah MR. Serum concentration of asprosin in new-onset type 2 diabetes. Diabetol Metab Syndr. (2020) 12:1–8. 10.1186/s13098-020-00564-w 32714446 PMC7376837

[22] Tung Y-T Chiang P-C Chen Y-L Chien Y-W. Effects of melatonin on lipid metabolism and circulating irisin in sprague-dawley rats with diet-induced obesity. Molecules. (2020) 25:3329. 10.3390/molecules25153329 32708001 PMC7436261

[23] Friedewald WT Levy RI Fredrickson DS. Estimation of the concentration of low-density lipoprotein cholesterol in plasma, without use of the preparative ultracentrifuge. Clin Chem. (1972) 18:499–502. 10.1093/CLINCHEM/18.6.499 4337382

[24] Miao Y Qin H Zhong Y Huang K Rao C. Novel adipokine asprosin modulates browning and adipogenesis in white adipose tissue. J Endocrinol. (2021) 249:83. 10.1530/JOE-20-0503 33705351 PMC8052515

[25] Marmonti E Busquets S Toledo M Ricci M Bria J Oliva F. Immobilization in diabetic rats results in altered glucose tolerance a model of reduced locomotion/activity in diabetes. JCSM Rapid Commun. (2018) 1:1–15. 10.1002/j.2617-1619.2018.tb00007.x25855820

[26] Bancroft JD Gamble M. Theory and Practice of Histological Techniques, 6th ed. London: Churchill Livingstone (2007).

[27] Landau S Everitt B. A Handbook of Statistical Analyses Using SPSS, 1st ed. Boca Raton, FL: CRC Press (2004).

[28] Khaled AA Abulfadle MD Rania RA Atia MD. Effect of oxytocin treatment on asprosin serum level and liver function changes in rats with streptozotocin-induced diabetes. Med J Cairo Univ. (2018) 86:4417–28. 10.21608/mjcu.2018.63143

[29] Blevins JE Graham JL Morton GJ Bales KL Schwartz MW Baskin DG. Chronic oxytocin administration inhibits food intake, increases energy expenditure, and produces weight loss in fructose-fed obese rhesus monkeys. Am J Physiol. (2015) 308:R431. 10.1152/ajpregu.00441.2014 25540103 PMC4346756

[30] Lawson EA Marengi DA DeSanti RL Holmes TM Schoenfeld DA Tolley CJ. Oxytocin reduces caloric intake in men. Obesity. (2015) 23:950. 10.1002/oby.21069 25865294 PMC4414748

[31] Elabd S Sabry I. Two birds with one stone: possible dual-role of oxytocin in the treatment of diabetes and osteoporosis. Front Endocrinol. (2015) 6:121. 10.3389/fendo.2015.00121 26322016 PMC4530313

[32] Akour A Violet K Nailya B Yasser B Randa N Dana Hyasat. Levels of metabolic markers in drug-naive prediabetic and type 2 diabetic patients. Crossmark. (2016) 54:163–70. 10.1007/s00592-016-0926-1 27752839

[33] Björkstrand E Eriksson M Uvnäs-Moberg K. Evidence of a peripheral and a central effect of oxytocin on pancreatic hormone release in rats. Neuroendocrinology. (1996) 63:377–83. 10.1159/000126978 8739893

[34] Ho JM Blevins JE. Coming full circle: contributions of central and peripheral oxytocin actions to energy balance. Endocrinology. (2013) 154:589–96. 10.1210/EN.2012-1751 23270805 PMC3548187

[35] Mok JK Makaronidis JM Batterham RL. The role of gut hormones in obesity. Curr Opin Endocr Metab Res. (2019) 4:4–13. 10.1016/j.coemr.2018.09.005

[36] Elnagar A El-Belbasi HI Rehan IF El-Dawy K. Asprosin: A novel biomarker of type 2 diabetes mellitus. Slov Vet Res. (2018) 55:333–47. Available online at: https://www.slovetres.si/index.php/VMHE/article/download/661/147

[37] Snider B Geiser A Yu XP Beebe EC Willency JA Qing K. Long-Acting and selective oxytocin peptide analogs show antidiabetic and antiobesity effects in male mice. J Endocrine Soc. (2019) 3:1423–44. 10.1210/js.2019-00004 31286109 PMC6608564

[38] Romere C Duerrschmid C Bournat J Constable P Jain M Xia F. Asprosin, a fasting-induced glucogenic protein hormone. Cell. (2016) 165:566–79. 10.1016/j.cell.2016.02.063 27087445 PMC4852710

[39] Mihailović M Živković M Jovanović JA Tolinački M Sinadinović M Rajić J. Oral administration of probiotic lactobacillus paraplantarum BGCG11 attenuates diabetes-induced liver and kidney damage in rats. J Funct Foods. (2017) 38:427–37. 10.1016/j.jff.2017.09.033

[40] Mohan S Khan D Moffett RC Irwin N Flatt P.R. Oxytocin is present in islets and plays a role in beta-cell function and survival. Peptides. (2018) 100:260–8. 10.1016/j.peptides.2017.12.019 29274352

[41] Jones AG Hattersley AT. The clinical utility of c-peptide measurement in the care of patients with diabetes. Diabet Med. (2013) 30:803. 10.1111/dme.12159 23413806 PMC3748788

[42] Eckertova M Ondrejcakova M Krskova K Zorad S Jezova D. Subchronic treatment of rats with oxytocin results in improved adipocyte differentiation and increased gene expression of factors involved in adipogenesis. Br J Pharmacol. (2011) 162:452. 10.1111/j.1476-5381.2010.01037.x 20846187 PMC3031065

[43] Mohamed NA Nassier OA. The antihyperglycaemic effect of the aqueous extract of origanium vulgare leaves in streptozotocin-induced diabetic rats. Jordan J Biol Sci. (2013) 6:31–8. 10.12816/0000256

[44] Kondeti VK Badri KR Maddirala DR Thur SKM Fatima SS Kasetti RB. Effect of pterocarpus santalinus bark, on blood glucose, serum lipids, plasma insulin and hepatic carbohydrate metabolic enzymes in streptozotocin-induced diabetic rats. Food Chem Toxicol. (2010) 48:1281–7. 10.1016/j.fct.2010.02.023 20178824

[45] Elabd C Cousin W Upadhyayula P Chen RY Chooljian MS Li J. Oxytocin is an age-specific circulating hormone that is necessary for muscle maintenance and regeneration. Nat Commun. (2014) 5:4082. 10.1038/ncomms5082 24915299 PMC4512838

[46] Nabi SA Kasetti RB Sirasanagandla S Tilak TK Kumar MVJ Rao CA. Antidiabetic and antihyperlipidemic activity of piper longum root aqueous extract in STZ induced diabetic rats. BMC Complement Altern Med. (2013) 13:37. 10.1186/1472-6882-13-37 23414307 PMC3583796

[47] Brites F Martin M Guillas I Kontush A. Antioxidative activity of high-density lipoprotein (hdl): mechanistic insights into potential clinical benefit. BBA Clin. (2017) 8:66. 10.1016/j.bbacli.2017.07.002 28936395 PMC5597817

[48] Düşünceli F Işeri SÖ Ercan F Gedik N Yegen C Yegen BÇ. Oxytocin alleviates hepatic ischemia–reperfusion injury in rats. Peptides. (2008) 29:1216–22. 10.1016/j.peptides.2008.02.010 18403049

[49] Koroglu P Senturk GE Yucel D Ozakpinar OB Uras F Arbak S. The Effect of Exogenous Oxytocin on Streptozotocin (STZ)-Induced Diabetic Adult Rat Testes. Peptides. (2015) 63:47–54. 10.1016/j.peptides.2014.10.012 25451466

[50] Petersson M Wiberg U Lundeberg T Uvnäs-Moberg K. Oxytocin decreases carrageenan induced inflammation in rats. Peptides. (2001) 22:1479–84. 10.1016/S0196-9781(01)00469-7 11514032

[51] Song Z Levin BE Stevens W Sladek CD. Supraoptic oxytocin and vasopressin neurons function as glucose and metabolic sensors. Physiol Reg J. Integ Comp Physiol. (2014) 306:447–56. 10.1152/ajpregu.00520.2013 24477542 PMC3962623

[52] Maejima Y Rita RS Santoso P Aoyama M Hiraoka Y Nishimori K. Nasal oxytocin administration reduces food intake without affecting locomotor activity and glycemia with c-Fos induction in limited brain areas. Neuroendocrinology. (2015) 101:35–44. 10.1159/000371636 25573626

[53] Elabd SK Sabry I Mohasseb M Algendy A. Oxytocin as A novel therapeutic option for type i diabetes and diabetic osteopathy. Endocr Regul. (2014) 48:87–102. 10.4149/endo_2014_02_87 24824804

[54] Gerich JE. Is reduced first-phase insulin release the earliest detectable abnormality in individuals destined to develop type 2 diabetes? Proc Diabet. (2002) 51 (Suppl. 1):S117–21. 10.2337/diabetes.51.2007.S117 11815469

[55] Hu Y Xu Y Zheng Y Kang Q Lou Z Liu Q. Increased plasma asprosin levels in patients with drug-naive anorexia nervosa. Eat Weight Disord. (2020) 26:313–21. 10.1007/s40519-020-00845-3 32026376

[56] Wang Y Qu H Xiong X Qiu Y Liao Y Chen Y. Plasma asprosin concentrations are increased in individuals with glucose dysregulation and correlated with insulin resistance and first-phase insulin secretion. Mediators Inflamm. (2018) 2018:9471583. 10.1155/2018/9471583 29743813 PMC5883982

[57] Li X Liao M Shen R Zhang L Hu H Wu J. Plasma asprosin levels are associated with glucose metabolism, lipid, and sex hormone profiles in females with metabolic-related diseases. Mediators Inflamm. (2018) 2018:7375294. 10.1155/2018/7375294 30524197 PMC6247534

[58] Davies GF Khandelwal RL Wu L Juurlink BH Roesler WJ. Inhibition of phosphoenolpyruvate carboxykinase (*PEPCK*) gene expression by troglitazone: a peroxisome proliferator-activated receptor-gamma (PPARgamma)-independent, antioxidant-related mechanism. Biochem Pharmacol. (2001) 62:1071–9.? 10.1016/S0006-2952(01)00764-X 11597575

[59] Quinn PG Yeagley D. Insulin regulation of *PEPCK* gene expression: a model for rapid and reversible modulation. Curr Drug Targ Immune Endocrine Metab Disord. (2005) 5:423–37.? 10.2174/156800805774912962 16375695

[60] Ramnanan CJ Edgerton DS Cherrington AD. The Role of insulin in the regulation of *PEPCK* and gluconeogenesis *in vivo*. US Endocrinology. (2010) 5:34–9. 10.17925/USE.2009.05.1.3419755527

[61] Jung UJ Lee MK Park YB Jeon SM Choi MS. Antihyperglycemic and antioxidant properties of caffeic acid in db/db mice. J Pharmacol Exp Ther. (2006) 318:476–83.? 10.1124/jpet.106.105163 16644902

[62] Martin WH Hoover DJ Armento SJ Stock IA McPherson RK Danley DE. Discovery of a human liver glycogen phosphorylase inhibitor that lowers blood glucose *in vivo*. Proc Natl Acad Sci USA. (1998) 95:1776–81.? 10.1073/pnas.95.4.1776 9465093 PMC19188

[63] Vanderbilt Medical Center. Granner Lab, PEPCK Research. Vanderbilt Medical Center (2001). Available online at: www.mc.vanderbilt.edu/root/vumc.php?site=granner&doc=119

[64] Burgess SC He T Yan Z Lindner J Sherry AD Malloy CR. Cytosolic phosphoenolpyruvate carboxykinase does not solely control the rate of hepatic gluconeogenesis in the intact mouse liver. Cell Metab. (2007) 5:313–20. 10.1016/j.cmet.2007.03.004 17403375 PMC2680089

[65] Méndez-Lucas A Duarte JA Sunny NE Satapati S He T Fu X. PEPCK-M expression in mouse liver potentiates, not replaces, PEPCK-C mediated gluconeogenesis. J Hepat. (2013) 59:105–13. 10.1016/j.jhep.2013.02.020 23466304 PMC3910155

[66] Vijayaraj P Muthukumar K Sabarirajan J Nachiappan V. Antihyperlipidemic activity of cassia auriculata flowers in triton WR 1339 induced hyperlipidemic rats. Exp Toxicol Pathol. (2013) 65:135–41. 10.1016/j.etp.2011.07.001 21852078

[67] Sabu MC Smitha K Kuttan R. Anti-Diabetic activity of green tea polyphenols and their role in reducing oxidative stress in experimental diabetes. J Ethnopharmacol. (2002) 83:109–16. 10.1016/S0378-8741(02)00217-9 12413715

[68] Vozarova B Stefan N Lindsay RS Saremi A Pratley RE Bogardus C. High alanine aminotransferase is associated with decreased hepatic insulin sensitivity and predicts the development of type 2 diabetes. Diabetes. (2002) 51:1889–95. 10.2337/diabetes.51.6.1889 12031978

[69] Moon JS Lee JE Yoon JS. Variation in serum creatinine level is correlated to risk of type 2 diabetes. Endocrinol Metab. (2013) 28:207. 10.3803/EnM.2013.28.3.207 24396680 PMC3811691

[70] Kim H Ahn Y. Role of peroxisome proliferator-activated receptor-γ in the glucose-sensing apparatus of liver and β-cells. Diabetes. (2004) 53:S60–. 10.2337/diabetes.53.2007.S60 14749267

[71] Gutkowska J Jankowski M. Oxytocin: old hormone, new drug. Pharmceuticals. (2009) 2:168–83. 10.3390/ph203168 27713231 PMC3978540

[72] Bhonde R Shukla RC Kanitkar M Shukla R Banerjee M Datar S. Isolated islets in diabetes research. Indian J Med Res. (2007) 125:425–40. Available online at: https://journals.lww.com/ijmr/Abstract/2007/25030/Isolated_islets_in_diabetes_research.18.aspx 17496366

[73] Panigrahy SK Bhatt R Kumar A. Reactive oxygen species: sources, consequences and targeted therapy in type 2 diabetes. J Drug Target. (2016) 25:93–101. 10.1080/1061186X.2016.1207650 27356044

